# Genome-Wide Patterns of *Arabidopsis* Gene Expression in Nature

**DOI:** 10.1371/journal.pgen.1002662

**Published:** 2012-04-19

**Authors:** Christina L. Richards, Ulises Rosas, Joshua Banta, Naeha Bhambhra, Michael D. Purugganan

**Affiliations:** 1 Department of Biology and Center for Genomics and Systems Biology, New York University, New York, New York, United States of America; 2 Department of Integrative Biology, University of South Florida, Tampa, Florida, United States of America; 3 Department of Biology, University of Texas at Tyler, Tyler, Texas, United States of America; Georgia Institute of Technology, United States of America

## Abstract

Organisms in the wild are subject to multiple, fluctuating environmental factors, and it is in complex natural environments that genetic regulatory networks actually function and evolve. We assessed genome-wide gene expression patterns in the wild in two natural accessions of the model plant *Arabidopsis thaliana* and examined the nature of transcriptional variation throughout its life cycle and gene expression correlations with natural environmental fluctuations. We grew plants in a natural field environment and measured genome-wide time-series gene expression from the plant shoot every three days, spanning the seedling to reproductive stages. We find that 15,352 genes were expressed in the *A. thaliana* shoot in the field, and accession and flowering status (vegetative versus flowering) were strong components of transcriptional variation in this plant. We identified between ∼110 and 190 time-varying gene expression clusters in the field, many of which were significantly overrepresented by genes regulated by abiotic and biotic environmental stresses. The two main principal components of vegetative shoot gene expression (PC^veg^) correlate to temperature and precipitation occurrence in the field. The largest PC^veg^ axes included thermoregulatory genes while the second major PC^veg^ was associated with precipitation and contained drought-responsive genes. By exposing *A. thaliana* to natural environments in an open field, we provide a framework for further understanding the genetic networks that are deployed in natural environments, and we connect plant molecular genetics in the laboratory to plant organismal ecology in the wild.

## Author Summary

Plants in the real world are continuously exposed to multiple environmental signals and must respond appropriately to the dynamic conditions found in nature. Environmental signals can fluctuate during an individual's life cycle with varying degrees of predictability, and complex natural environments are where gene activity evolves. We grew two natural accessions of the model plant *Arabidopsis thaliana* in an open field in New York in the spring and examined genome-wide gene expression patterns in the wild. We find nearly 200 gene expression clusters in these field-grown plants, and many of these clusters were enriched in genes that had previously been shown to be associated with expression under various abiotic or biotic environmental stress conditions. Two major principal components of gene expression were associated with environmental fluctuations in temperature and rainfall, and we identified several genes (such as the thermoregulatory nucleosome occupancy gene *ARP6* and the drought-sensitive hormone biosynthetic gene *AAO3*) that could be found in these principal components. By exploring genome-wide gene expression in plants in the wild, we were able to connect mechanistic aspects of plant molecular biology with ecological responses in nature and to begin to understand how organisms behave and adapt in their natural environments.

## Introduction

Organisms in the real world are continuously exposed to multiple environmental signals and must respond appropriately to dynamic, fluctuating conditions found in nature [1]. Dynamic environmental signals can differ spatially and temporally during an individual's life cycle with different degrees of predictability, and it is in the context of complex natural environments that genetic regulatory networks actually function and evolve. The response to dynamic fluctuating environments is particularly critical for sessile organisms such as plants that cannot react behaviorally to adverse conditions but must respond by modulating development and physiology to cope with changing conditions [2], [3].

Temperature, water levels, biotic interactions and resource availability are just some key environmental conditions that cue organismal responses, and there have been significant advances in dissecting how these and other ecological signals are transduced by the organism to appropriate gene expression levels that may ultimately determine phenotypes [2]–[9]. With very few exceptions, however, studies on molecular genetic responses to the environment are undertaken in homogenous controlled laboratory conditions. The natural world, in contrast, is anything but controlled, and understanding how genes are regulated in natural ecological settings in the midst of fluctuating environmental signals remains a key objective of the new fields of ecological genomics and systems biology [6].

*Arabidopsis thaliana* has become one of the key plant model species, not only for studies of genetics and development but for ecology and evolution as well [10]–[12]. This species is a weedy annual plant, occupying disturbed habitats such as the margins of agricultural fields as well as natural ruderal environments. It is native to Europe and Central Asia [13], but has extended its range to include eastern and northwestern portions of the United States [13], [14]. A large proportion of natural populations adopt the spring annual strategy, with germination and flowering in spring [15]. *Arabidopsis thaliana* displays a wide range of ecological relationships, including within- and between-species interactions and adaptations to abiotic environments. It responds physiologically and developmentally to a variety of environmental cues, including light, daylength, vernalization, nutrient and water levels [10], [11], [15], and can be affected by bacterial and fungal pathogens [16], and by insect herbivory [17].

Despite the role of *A. thaliana* as a model plant system, remarkably little is known about the phenotypic range and performance of this species in the wild. Focusing on studies in natural field conditions may thus provide opportunities for a more comprehensive view, not only of ecological processes in this species, but also of development and physiology not possible in controlled laboratory experimentation. Indeed, a few field studies of *A. thaliana* have begun to shed light on the ecological genetics and natural selection in this species in field conditions [18]. Other field studies have looked at natural selection for and costs of herbivory defense traits [19]–[21], seasonal germination timing [22], fitness costs of *R* gene polymorphisms [23], the role of epistasis in fitness-related traits [24], and the genetic architecture of flowering time [25], [26].

Although it is clear that organismal phenotypes and the genetic architecture of various traits differ between controlled laboratory and field conditions, the extent to which patterns of gene expression is modulated in the wild is not at all understood. There are a large number of global gene expression studies in *A. thaliana* [27]–[31], and some notable investigations include a comprehensive developmental expression map [29], a cell-specific expression atlas of the root [28] and studies of circadian clock gene regulation [27].

All these studies, however, were undertaken in controlled laboratory conditions. Global gene expression studies of plant species in field conditions [32]–[42] demonstrate that there are significant transcription level differences between controlled and field growth conditions. A study in *A. thaliana* used responses to increased CO_2_ and ozone levels in Free Air CO_2_ Enrichment (FACE) environment [32]. From this study, >1,000 transcripts were either up- or down-regulated between controlled versus field ambient conditions compared with high vs. low CO_2_ or ozone levels, and there was a preponderance of genes associated with general defense reactions, secondary metabolism, redox control, energy provision, protein turnover, signaling and transcription [32]. More detailed experimental studies have also managed to connect specific genes with phenotypes; for example, seasonal flowering time response in *A. halleri* in the wild has been shown to be associated with expression of the *FLC* gene [43].

To contribute to our understanding of the ecological genomics and systems biology of plants in the wild, we determined genome-wide gene expression profiles in the shoot throughout the life cycle of the model plant *A. thaliana* under natural field conditions. We chose two distinct *A. thaliana* accessions Bayreuth-0 or Bay-0 (originally from a fallow field in Bayreuth, Germany) and Shakdara or Sha (from a mountainous site at Pamiro-Alay, Tajikistan) because there are genetic [44] and genomic resources [45], [46] in these accessions that can be further used to dissect molecular mechanisms of environmental response. Our study allowed us to identify genes that significantly vary across the spring life cycle of these two accessions and determine patterns of transcriptional co-regulation in field conditions. We found that in addition to accession and flowering stage, temperature and precipitation appear to be correlated with large-scale gene expression patterns in the field, and a large number of co-expressed gene clusters are enriched in loci responsive to several abiotic and biotic stresses. Our results suggest that stress-responsive loci are not only adaptive for extreme environments, but are deployed during the life cycle of *A. thaliana* to deal with normally fluctuating environments.

## Results

### A large fraction of protein-coding genes are expressed in the *Arabidopsis* shoot in the wild

**Figure 1:**
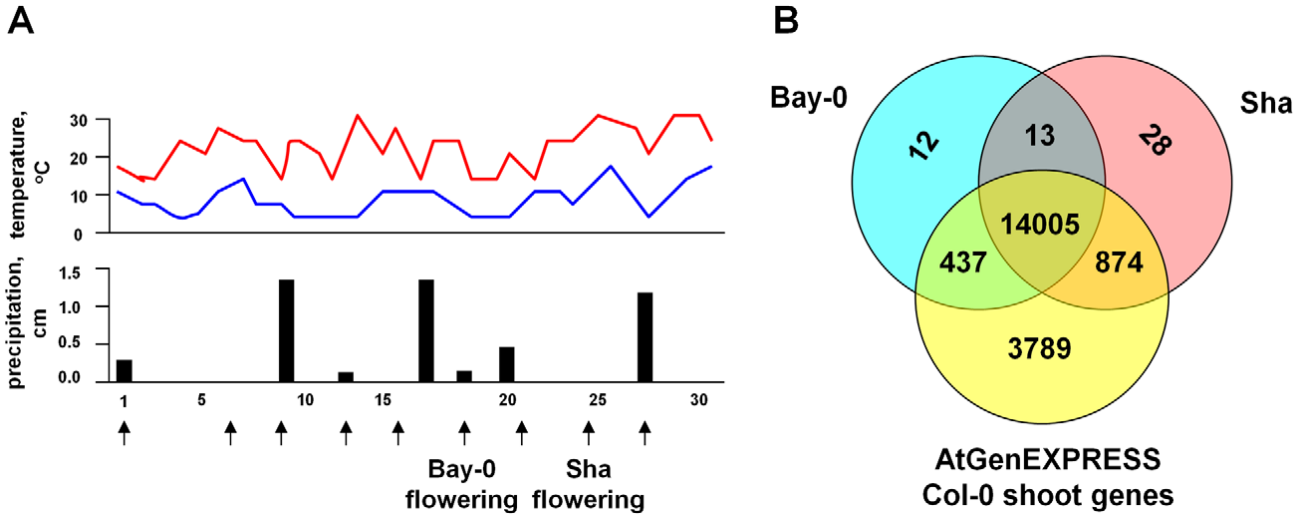
Field environmental conditions and total gene expression in *A. thaliana*. (A) The top graph shows the daily maximum (red) and minimum temperatures (blue), and the lower graph shows the daily levels of precipitation in the field during the experiment. The sampling days (given as days after germination) are shown in the bottom. The arrows indicate the sampling points, and the onset of flowering for Bay-0 and Sha in the experiment are shown. (B) The total number of genes expressed in Bay-0 and Sha in the field, compared to those observed for Col-0 shoot tissue in the ATGenExpress data set [29].

We assayed RNA from replicate pools of shoot samples for genome-wide gene expression of *A. thaliana* across its life cycle in the complex and natural conditions of the field using the Affymetrix ATH1 gene chip. The field experiment spanned the seedling (∼4-leaf) stage to flowering in the late spring of 2008 at the Cold Spring Harbor Laboratory field site (see Figure 1). During this experimental period, daily temperatures ranged from a mean low of 8.7°C to a mean high of 23.7°C. Of the 30 days that the plants were outside, it rained only 8 days, with precipitation levels during these days ranging from 2.5 to 31.8 mm.

Despite the possibility of environmental heterogeneity in this outdoor field site, the replicates for the eight Bay-0 and ten Sha samples produced very similar results (mean pairwise correlation = 0.98, see Figure S1), which is comparable to replication quality observed in controlled laboratory experiments [29]. Genes were designated as expressed if they were observed in all three replicates at a timepoint by the Affymetrix Microarray Suite 5 (MAS 5) algorithm [47], and we found that 47% to 58% of genes in Bay-0 and 45% to 61% in Sha were expressed at each timepoint. In total, we found that ∼67% of genes were expressed in at least one accession for at least one time point (see Figure S2).

We compared our results to those observed in the ATGenExpress [29] data set. In total, we detected 15,369 genes in at least one accession (∼67% of genes), which is less than the 19,105 genes detected in the 48 comparable vegetative and flower tissue samples from wild type Col-0 in the ATGenExpress data set. Only 53 genes that were not found in the Col-0 shoot expression atlas showed expression in the Bay-0/Sha field dataset. The reduced number of detected genes in our experiment could reflect the fact that the ATGenExpress is a compendium of experiments done under multiple experimental conditions, some of which may not be relevant to the field conditions under which we conducted our study. Moreover, the ATGenExpress analysis has greater power to detect expression level differences given the larger sample sizes in that study [29].

The majority of the genes that were expressed in Col-0 overlapped with both Bay-0 and Sha samples (14,005 genes) (see Figure 1B). Across the Bay-0 and Sha field samples, expression for 7,459 genes (∼33%) were undetected, of which more than half were unannotated loci (4,626 genes in Bay-0 and 4,415 in Sha, FDR<0.05; see Figure S3). Twelve other GO terms were significantly over-represented among these unexpressed genes across the two accessions, including defense response and transcriptional regulation genes.

**Figure 2:**
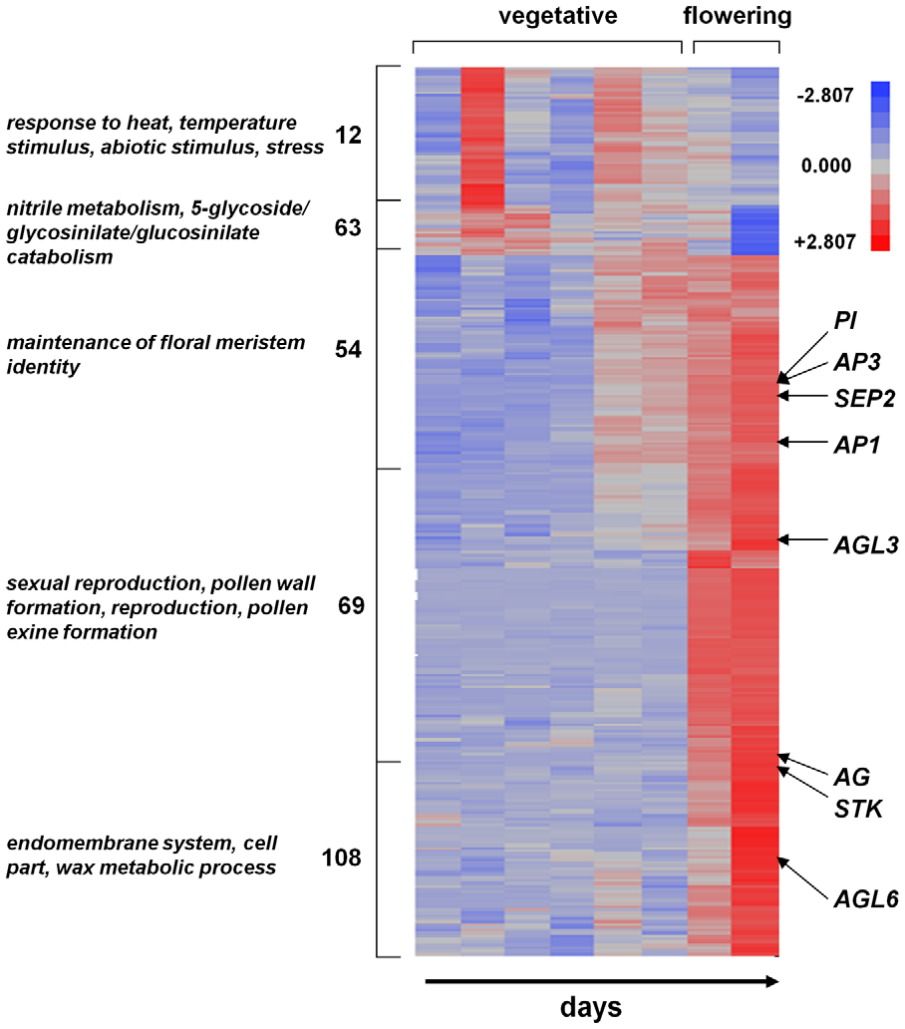
Heat map for Bay-0 genes that show differential expression during flowering. The numbers listed to the left indicate the cluster number identified in the silhouette analyses. The four most significant (i.e., lowest *p* values) GO term categories enriched for each cluster are shown on the left. The vegetative and flowering samples are indicated at the top, and the rows corresponding to various flower development genes are shown. Several developmental genes associated with bolting and flower development are highlighted. Scale: from brightest blue equals most down-regulated to brightest red equals most up-regulated.

The flowering of *A. thaliana* in the field provides an internal validation of the observed gene expression patterns, since several genes have been identified that are associated with flowering and flower development. We created a heat map of groups of unique gene expression patterns (defined by cluster analysis, description of analysis below) for which more than 50% of the variance was explained by flowering status in both Bay-0 and Sha (see Figure 2). As expected, these clusters contain several floral developmental genes, including *AP1* [48], *AP3* [49], *PI* [50], *AG* [51], *STK* [52], and *SEP2* [53]. The observation that the expression of these floral genes increase upon flowering provides confidence that our results in the field are consistent with expectations based on previous developmental genetic studies [29].

### Strong differentiation in genome-wide gene expression patterns between *A. thaliana* accessions

While we expected to find that the flowering states of *A. thaliana* (vegetative vs. flowering) are transcriptionally distinct, we also found that natural genotypic differences between accessions are an equally important component of genome-wide differences in gene expression patterns under field conditions. We ran a principal variance components analysis (PVCA) [54] of the ∼22,800 genes expressed across the combined Bay-0 and Sha data set to examine whether gene expression is explained by accession or flowering status (vegetative or flowering). This approach first reduces the dimensionality of the data set with principal components analysis (PCA), and then computes variance components by fitting a mixed linear model to each principal component (PC*i* = accession+flowering status+accession-by-flowering status+error). For each factor in the model, the variance components are averaged across all of the principal components, but weighted by the eigenvalues for the corresponding principal component.

**Figure 3:**
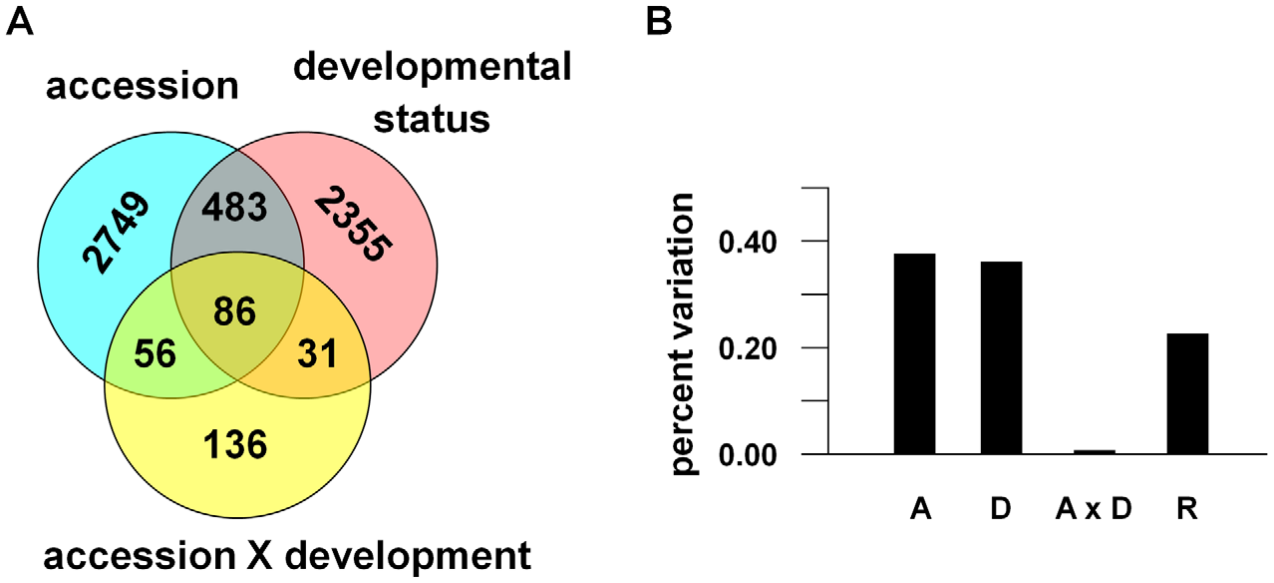
Components of transcriptional variance in *A. thaliana* in the field. (A) The number of genes that significantly differ between Bay and Sha accessions, flowering status, and that show an accession-by-flowering status (development) interaction. (B) The percent of the transcriptional variance explained by these factors.

Principal component 1 (PC1^all^) explained 18% of the variation in genome-wide gene expression and clearly distinguished the two accessions, while vegetative vs. flowering states were demarcated along PC2^all^ (16%) and PC3^all^ (10%). The mixed linear model of the principal components attributed approximately equal amount of the transcriptional variance to accession (39%) and flowering status (38%; see Figure 3). We modeled the effects of accession and flowering status on gene expressions with a mixed model analysis of variance (ANOVA): gene expression = accession+flowering status+accession-by-flowering status+error. The analysis indicated that the two accessions differed significantly in 3,344 genes (∼14% of the transcripts; see Figure 3), which is within the range previously found for several accessions [55]. This apparent discrepancy between the number of genes that significantly vary between accessions and the fraction of transcriptional variance explained by accession indicates that a small fraction of genes can explain a large amount of expression variation, which has been shown in other comparisons between accessions [56].

In addition, 2,955 genes (13% of transcripts) significantly differed between vegetative and bolting shoots, 569 of these were also different between accessions, and 117 of these also showed a significant interaction effect between accession and flowering status (see Figure 3). The overall amount of variance explained by the interaction term was very small (<0.01%; only 309 genes total). These results suggest that while the flowering states of *A. thaliana* (vegetative vs. flowering) are transcriptionally distinct, natural genotypic differences between accessions show equally significant genome-wide differences in gene expression patterns under field conditions.

A gene ontology (GO) enrichment analysis on the combined data for the main effects of accession, flowering status and the interaction of these two terms showed accession differences are enriched for genes in the sulfate assimilation, glucosinolate and glutathione biosynthesis pathways, while unannotated and translation genes were underrepresented (FDR q<0.05, see Table S1). There are 21 GO categories overrepresented between vegetative and flowering states, including genes that are associated with development, pollen exine formation, and sexual reproduction.

**Figure 4:**
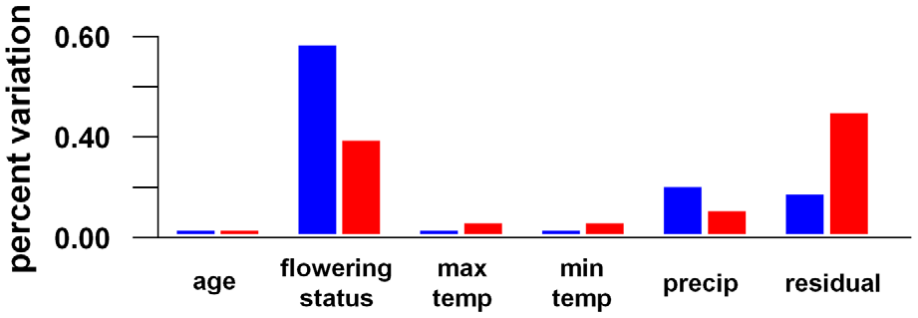
Environmental components of transcriptional variance in *A. thaliana* in the field. The blue indicates Bay-0 and the red Sha.

Using the same PVCA and mixed model ANOVA approach, we also looked at global trends in gene expression observed within each of the two accessions by examining how variation in genome-wide transcription levels is explained by various environmental factors. We examined the effects of age, flowering status (i.e., vegetative vs. flowering), minimum and maximum daily temperatures, and daily precipitation, using the mixed linear model: gene expression = age+flowering status+minimum temperature+maximum temperature+precipitation+error (see Figure 4).

Temperature and precipitation were the only environmental measurements we were able to obtain from the field site. Another environmental variable was daylength, but this was correlated with age (i.e., increasing daylength during the field experiment). We found weak correlations between the other environmental features during the field study: maximum and minimum daily temperatures (r^2^ = 0.17; p<0.007), maximum temperature and precipitation (r^2^ = 0.26; p<0.001), and minimum temperature and precipitation (r^2^ = 0; p<0.77).

Looking at each accession separately, flowering status explained more than 50% of the variance in expression across the genome for Bay-0 and over 30% of the variance for Sha. The only environmental variable that explained a substantial portion of transcriptional variance was precipitation (23% in Bay-0, 13% in Sha); plant age, minimum and maximum temperature explained negligible levels of variance (see Figure 4).

### Significant variation in gene expression across the *A. thaliana* life cycle in the field

Genes whose expression levels are variable in time across the life cycle in the field are of great interest, since they may provide insights on the transcriptomic response to development and environment (i.e., transcriptomic plasticity). An alternative approach to ANOVA that may be more appropriate for significance testing of time course microarray data fits a cubic spline to gene expression levels across time and tests for significant deviation from an invariant gene expression pattern [57], [58]. Using this approach, 12,599 genes in Bay-0 and 15,824 genes in Sha (FDR of q<0.05) display significant variation in time. GO analyses of these genes found enrichment for genes associated with metabolism, microtubule-based processes, heat and stress response, and transport processes. While the larger number of time-variable genes in Sha could reflect higher developmental and environmental transcriptomic plasticity (i.e. Bay-0 might be more robust), it more likely reflects the larger number of time-points sampled in Sha and its consequent exposure to a wider range of environments due to later flowering.

### Several co-expressed gene clusters in the wild are enriched for abiotic and biotic stress-inducible genes

A major goal of this study is to identify genes and gene clusters that may be associated with fluctuations in natural environmental conditions in the field. One approach we took was to identify gene clusters in the expression profiles, and correlate these with environmental factors and previously published microarray studies of abiotic/biotic stress responses.

**Figure 5:**
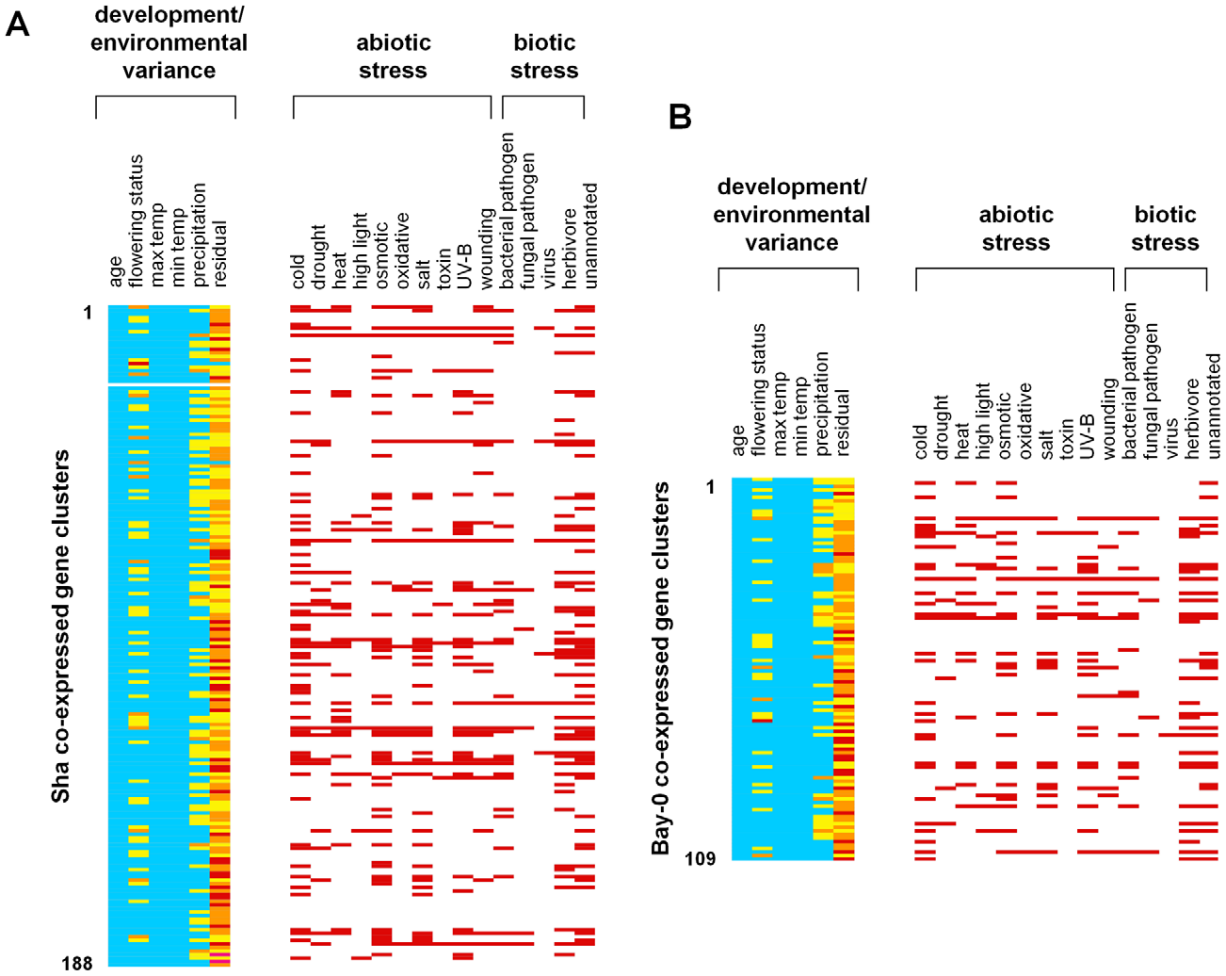
Gene expression clusters in *A. thaliana* field transcriptomes. Silhouette analysis defined (A) 188 clusters in Sha and (B) 109 clusters in Bay-0. Each row indicates a specific cluster. The components of the transcriptional variance explained by environmental (precipitation, maximum and minimum daily temperatures) and developmental factors (age and flowering status) are shown. The percent of the transcriptional variance explained by a factor is shown in color (blue, <25%; yellow, between 25–50%; orange, between 50–75%; red, >75%). We also examined each cluster for enrichment of genes shown to be differentially expressed in previously-studied transcriptome analyses. A red bar indicates that the cluster is enriched in genes differentially expressed under that stress condition.

We identified distinct co-expressed gene clusters in the life cycle of these accessions in the wild. We used the mean expression values of significantly time-variable genes found at an FDR of q<0.01 to isolate strong signatures of response to environmental factors. Under this criterion, we analyzed 3,827 genes in Bay-0 and 8,215 genes in Sha using the silhouette method [59], to identify 109 co-expressed gene clusters in Bay-0 and 188 clusters in Sha (see Figure 5). This number of clusters was similar to that identified by K-means clustering with a correlation of between 0.75 and 0.8 in Bay-0 (105 and 163 distinct clusters) and between 0.7 and 0.75 in Sha (169 and 277 distinct clusters).

For each cluster, we used the PVCA approach to fit the mixed linear model: gene expression = age+flowering status+minimum temperature+maximum temperature+precipitation+error. There were several clusters that showed >50% of the variance explained by flowering status, although no cluster showed transcriptional effects due to plant age. Among the environmental responses, there were several clusters for which more than 50% of the variation was explained by precipitation – indicating up- or down-regulation of genes under high precipitation (>25 mm but not at <10 mm). While several clusters showed a small percentage of the variance explained by either minimum or maximum daily temperature, no cluster showed more than 50% of the variance explained by these factors suggesting that the genes responding specifically to temperature may be more dispersed across the structure of clusters we identified. We also compared the genes in these clusters to published microarray studies on gene expression under known environmental stresses. These studies encompass various abiotic and biotic stresses that affect gene expression, including high light [60], cold, drought, heat, osmotic stress, oxidative stress, salt, genotoxins, UV-B exposure, wounding [61], and infection by RNA virus [62], [63], bacterial pathogens, fungi and herbivores [64]. Combining these data sets, more than half of the genes on the ATH1 array were associated with at least one stress (13,153 of 22,800 genes). Enrichment in specific clusters for genes associated with differential expression in each of these stress responses could provide clues about the ecological factors that drive the underlying field expression patterns.

An intriguing result of the stress annotation analysis was that many gene clusters appeared to contain genes that responded to multiple laboratory stress treatments. For example, six co-expressed gene clusters in Bay-0 and ten clusters in Sha appeared to contain genes that were responsive to nine or more stresses (see Figure 5), suggesting that these loci were associated with generalized stress responses. These include several genes encoding heat-shock proteins (e.g, *Hsp70*, *Hsp101*, *Hsp17.6*), the cold- and ABA inducible gene *kin1*, cold-regulated genes *cor15a* and *b*, and the stress-responsive *LT16* and *sti1*-like protein-coding loci. These general stress response clusters included both biotic and abiotic stresses in all but one of the Bay and one of the Sha clusters, which were enriched for only abiotic stresses. In general, we found strong overlap between response to abiotic and biotic stresses. For example in Bay, 28 clusters were enriched for both types of stress, while 15 were enriched for only abiotic stresses and five only for biotic stresses. In Sha, 42 clusters were enriched for both types of stress, with 36 clusters enriched only for abiotic stresses and 11 clusters enriched only for biotic stresses.

On the other hand some clusters were enriched for response to only one specific stress: for example nine Sha clusters were enriched only for response to cold, four clusters were enriched only for response to osmotic stress, two clusters were enriched only for response to UV-B radiation, and five clusters were enriched only for response to herbivory (see Figure 5).

### Principal component analysis of vegetative stage gene expression in the wild

Our analysis looked at expression across both vegetative and reproductive stages of the life cycle. Given the strong effect of flowering status on gene expression patterns, we also undertook a principal component analysis of gene expression on the vegetative stages in the field, and examined correlations with environmental conditions of the PC scores. A subset of genes significantly expressed in both accessions across vegetative stages, were analyzed to minimize the differences due to flowering stage (i.e. PC1^all^ = 17.9% of the variance), and accession (PC2^all^ = 15.6% of the variance). Thus a total of 14 samples with three replicates each (6 samples from Bay-0 and 8 samples from Sha) and 8,954 genes were analyzed by principal component analysis.

**Figure 6:**
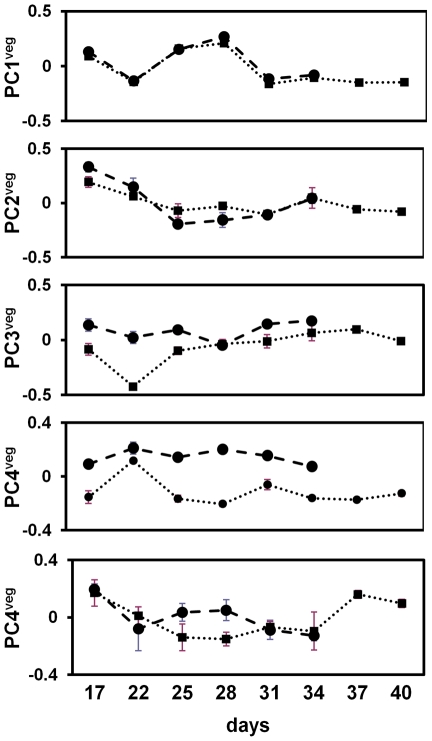
Vegetative stage principal component analyses of *A. thaliana* field transcriptome. The first five principal components are indicated. Principal component scores are on the y-axis and sampling days on the x-axis. The dashed line gives the PC score for Bay-0 and the dotted line for Sha. At each sampling day, the mean PC score across the three replicates are given for Bay-0 (circles) and Sha (squares), with standard error bars (+/− 1 SE).

Ten vegetative stage principal components (PC^veg^) captured ∼70% of the variation between accessions and across time points. To visualize the trends of the variation, the mean PC^veg^ values for each accession were plotted against their corresponding time points (see Figure 6). Only the first five vegetative stage principal components are shown as they captured more than 50% of the transcriptional variation. PC1^veg^, PC2^veg^ and PC5^veg^ showed no significant differences between Bay-0 and Sha. In contrast PC3^veg^ and PC4^veg^ showed differences across most time points between accessions, suggesting that these principal components still capture some of the expression differences due to genetic background. This indicates that the subset of genes significantly expressed in vegetative stages was not sufficient to account for accession effects (i.e. PC1^all^), but it uncovers other trends of gene expression variation that have an environmental basis.

To assess possible environmental or developmental associations with these principal components of gene expression, we used multivariate regression to regress PC^veg^ values on measured variables (see Table 1). Minimum daily temperature was not considered in the model because it was co-linear with maximum daily temperature. PC1^veg^ was significantly negatively correlated with maximum daily temperatures (β = −0.025, p<0.001) and marginally significant with RLN (β = 0.025, p<0.049), explaining a significantly large proportion of the variance (adjusted r^2^ = 0.87, p<0.0001). PC2^veg^ was significantly correlated to daily precipitation levels (β = −0.17, p<0.001) and age (β = −0.014, p<0.001), which explained significant proportions of the variance (adjusted r^2^ = 0.53, p<0.0001). The third principal component of global vegetative gene expression did not show a significant linear correlation with any of the environmental factors or plant development. These results predict that genes correlated to PC1^veg^ might be related to temperature responses, whereas those that correlate to PC2^veg^ might be related to water availability/drought responses.

**Table 1:** Characteristics of the major vegetative stage principal components of *A. thaliana* genome-wide gene expression in the field.

	GO Term enrichment	Number of genes	p	Environmental correlation
**PC1^veg^**	response to temperature stimulus	22	6.4×10^−5^	TMAX***RLN*
	response to abiotic stimulus	40	6.7×10^−5^	
	response to heat	12	4.0×10^−4^	
	response to stress	56	4.9×10^−4^	
	response to stimulus	81	1.8×10^−3^	
	biosynthetic process	95	9.9×10^−3^	
**PC2^veg^**	response to chemical stimulus	44	5.6×10^−3^	PPT**Age**
**PC3^veg^**	Unannotated	99	4.7×10^−5^	
**PC4^veg^**	None			RLN**Age**
**PC5^veg^**	fatty acid metabolic process	13	7.1×10^−3^	

TMAX: Maximum daily temperature, PPT: daily precipitation; RLN: rosette leaf number.

***: p<0.001;

**: p<0.01.

*: p<0.05.

We examined the gene sets for gene ontology (GO) term enrichments (p≤0.01; hypergeometric distribution) to identify significantly over-represented functional gene classes in Virtual Plant 1.0 [65] (see Table 1). First, the entire set of 8,954 genes that showed significant time series expression differences in the vegetative stages, was analysed for GO term enrichment; these showed *cell part, membrane, plasma membrane, response to chemical stimulus, response to stimulus, response to abiotic stimulus, intracellular part, membrane bounded organelle*, and *intracellular membrane-bounded organelle* as over-represented gene ontology categories.

In order to understand what gene functions are associated with each principal component, we conducted GO term enrichment analyses for gene sets associated with each PC^veg^. We selected genes that showed extreme PC loadings for each of the PC^veg^ axes (upper and lower 2.5 percent of the quantile distributions); thus we selected the 5% of the genes that showed the best correlation to each PC^veg^. The results of the analyses on each vegetative stage principal component showed that genes strongly associated with PC1^veg^ are mainly from the GO categories *response t*o *temperature stimulus*, *response to abiotic stimulus*, *response to heat*, *response to stress*, *response to stimulus*, which is consistent with the observation of maximum daily temperature and temperature fluctuation explaining a significant proportion of the variance in this principal component of expression. Genes with strong loadings on PC2^veg^ are orthogonal (uncorrelated) to genes in PC1^veg^, and were over-represented in the GO categories *response to chemical stimulus* genes, which might reflect growth and stress responses regulated by common hormone signaling cascades [66] rather than the enrichment in the 8954 genes dataset. Genes strongly associated with PC3^veg^ are typically *unannotated* genes, but 32 are transposable elements. PC5^veg^ was associated with genes related to fatty acid metabolism [67], while genes with high loadings in PC4^veg^ did not show overrepresentation from any GO category. Gene lists for PC1^veg^ and PC2^veg^ (2.5 and 5% of quantiles) are shown in the Tables S2, S3, S4, S5.

### Expression of the temperature response regulatory network in the vegetative stage in the wild

It has recently been shown that approximately half of the transcript responses to ambient temperature in *A. thaliana* are regulated by the chromatin remodeling gene *ARP6* [68]. This gene, formerly known as *ESD1*, encodes a subunit of the SWR1 complex required for insertion of the alternative histone H2A.Z into nucleosomes [69], [70]. *ARP6* regulates global response to ambient temperature in part by modulating nucleosome occupancy of H2A.Z [68].

**Figure 7:**
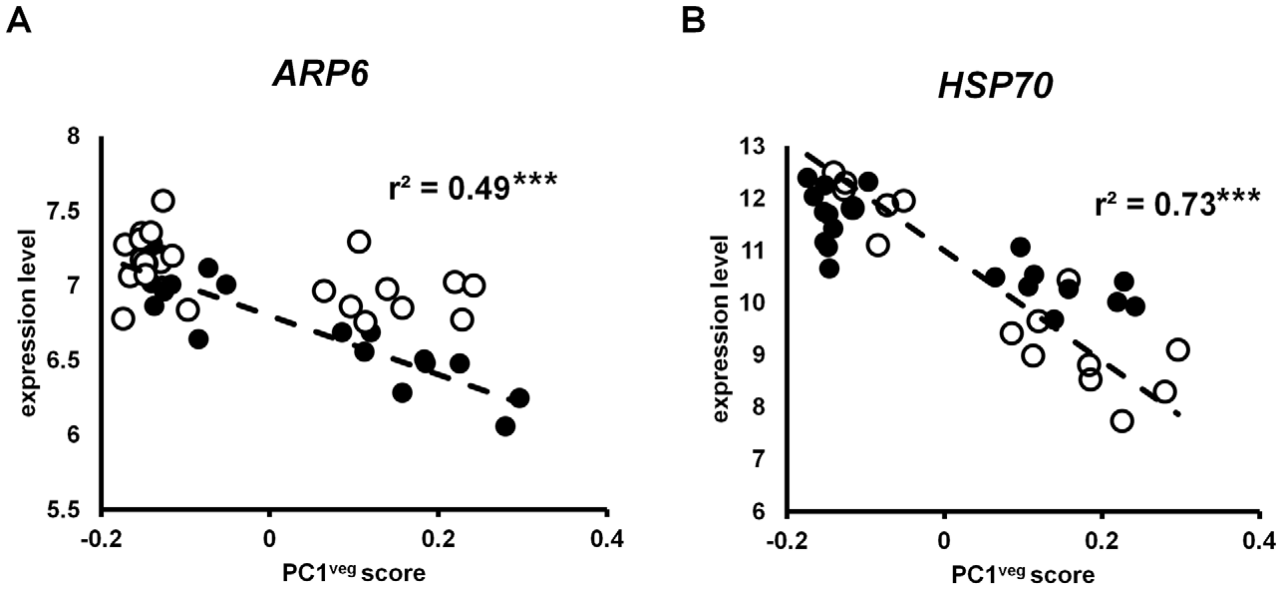
Gene expression in *A. thaliana* under field conditions correlated with PC1^veg^. Each point is the LS mean expression level of (A) *ARP6* and (B) *HSP70* in Bay-0 (open circles) and Sha (closed circles) for each time point. The dashed line shows the linear regression through the data. *** = p<0.001.

The *ARP6* gene was associated with PC1^veg^, and its expression was significantly correlated with this principal component (r^2^ = 0.49, p<0.001; see Figure 7), consistent with PC1^veg^ being correlated with temperatures in the field. One other temperature-regulated gene is the heat shock protein HSP70 [71], which is also regulated by *ARP6* [68]. Like *ARP6*, the expression of the *HSP70* gene was significantly correlated with PC1^veg^ (r^2^ = 0.73, p<0.001; see Figure 7).

Our results and those of Kumar and Wigge [68] both suggest that *ARP6* may regulate global temperature responses in the field by controlling nucleosome dynamics. We identified other genes that may be co-regulated with *ARP6* by finding loci whose expression in the field matched the absolute expression pattern of *ARP6* in our data, using Pavlidis template matching [72]. Previous studies have shown that temperature explains 47% of the variation in *ARP6* expression [r^2^ = 0.47; 68]. We used this threshold in our template matching analysis, and found that out of the 8,954 genes in our analysis, ∼40% (3,583 genes) in Bay-0 and ∼24% (2,118 genes) in Sha displayed expression profiles that were correlated to the *ARP6* field expression template at this threshold cut-off. These are consistent with previous studies that suggest that *ARP6* can control responses to temperature for a large number (∼5,000 genes) in *A. thaliana* [68].

To gain further understanding about the functions of these genes that are co-regulated with *ARP6*, we looked for over-represented GO term categories. One GO term that was enriched was *response to heat* with 47 genes in Bay-0 (p<10^−6^), and 27 genes in Sha (p<0.0003). The other enriched category was *unannotated genes*, with 573 genes in Bay-0 (p<10^−8^) and 339 genes in Sha (p<0.00015), out of which 67 and 47 genes respectively are transposable elements. The latter suggests that *ARP6* activity in the wild might be regulating other functions still to be described, and that transposable element activity may be triggered in part by environmental temperature fluctuations.

### ABA hormone signaling gene expression is associated with daily precipitation levels in pre-bolting plants

The second global vegetative principal component (PC2^veg^) was correlated to precipitation and fluctuating temperatures, and was significantly associated with genes involved in *chemical stimulus response* (p<0.007). Although among the genes associated with PC2^veg^ were those that are linked to auxin, cytokinin, gibberellic acid and brassinosteroid hormones, genes involved in the hormone abscisic acid (ABA) made up the largest fraction of hormone-associated loci in this principal component. This is noteworthy since ABA synthesis and signaling are known to mediate stress responses to water availability and osmotic stress, including drought and salinity stress responses, and PC2^veg^ is significantly correlated with daily precipitation levels (see Table 1).

**Figure 8:**
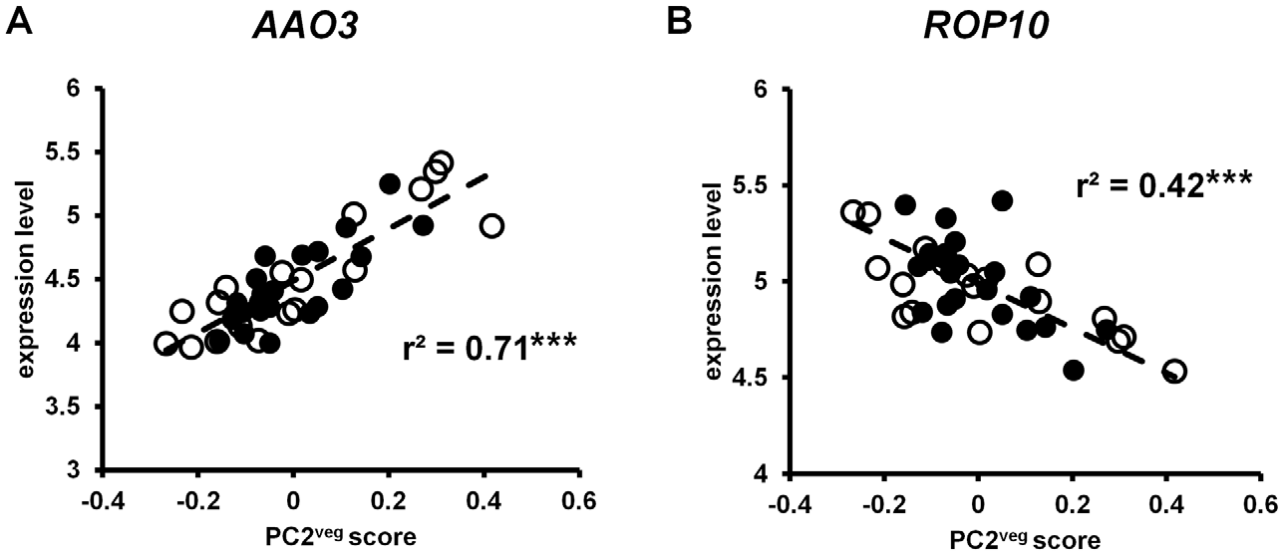
Gene expression in *A. thaliana* under field conditions correlated with PC2^veg^. Each point is the LS mean expression level of (A) *ROP10* and (B) *AAO3* in Bay-0 (open circles) and Sha (closed circles) for each timepoint. The dashed line shows the linear regression through the data. *** = p<0.001.

Among the ABA-associated genes significantly associated with PC2^veg^ was *ROP10*, which encodes a plasma membrane-bound rho-related GTPase protein that negatively regulates ABA responses [73], [74]. *ROP10* expression was significantly correlated with PC2^veg^ (r^2^ = 0.42, p<0.001; see Figure 8). To identify other genes that are co-regulated with *ROP10*, we obtained the genes that matched its absolute expression pattern using Pavlidis template matching. Unlike the temperature response analysis, we did not use previous data to guide our choice of correlation coefficient; we arbitrarily used a correlation coefficient of 0.7 in this analysis. Using this criterion, we identified many more genes co-regulated with *ROP10* in Bay-0 (273 genes) than in Sha (18 genes). Consistent with our finding of ABA hormone associated genes in PC2^veg^, and the significant correlation of this vegetative state principal component with daily precipitation levels, we found two genes in Bay-0 (At1g52080 and At5g61820) and one gene in Sha (At1g01470) that appear to be regulated by ABA levels [74]. Other ABA-associated genes correlated with PC2^veg^ include *AAO3* (r^2^ = 0.71, p<0.001; see Figure 8), which encodes an enzyme that catalyzes the last step of ABA biosynthesis in leaves [75], and *P5CS2* (r^2^ = 0.55, p<0.001) which encodes the rate-limiting enzymes for ABA-associated accumulation of proline under water stress [76], [77].

## Discussion

We have determined genome-wide expression profiles throughout the life cycle of the model plant *A. thaliana* under ecological field conditions to examine the nature of the transcriptome under the complex environment of a natural climatic season. Our analysis indicates that a majority of the genes in the *A. thaliana* genome show significant changes in gene expression throughout its life cycle in the field, and that accession is an important component of transcriptional variation among individuals. There are also clear effects of flowering status, as the onset of flowering not surprisingly leads to large-scale changes in transcriptional patterns in the *A. thaliana* shoot, with several genes associated with shoot bolting and floral development increasing in expression (see Figure 2). Despite the complexity in natural environments, transcriptional patterns are clearly organized into ∼100–200 co-expressed gene clusters in our *A. thaliana* accessions. Genetic studies have identified genetic networks that underlie plant responses to abiotic stress, including networks associated with drought responses [78], heat stress [79], [80] and cold responses [81]. Many of these networks contain genes that responded to multiple, laboratory-induced environmental stresses that have been identified by previous microarray studies in plants, including transcriptional responses to temperature [81]–[83] drought [61], [82], salt stress [83], metals [84], [85], nutrients [86], [87] and biotic challenges [88], [89]. Indeed, many of our inferred field gene expression clusters contain genes responsive to multiple environmental factors (see Figure 5), suggesting that these clusters may be responding to the complex conditions in field settings. It should be noted that the spring field environment in which our plants grew were not extreme in either temperature or precipitation levels, and our findings indicate that previously described stress genes may be associated simply with responses to normal environmental fluctuations that plants generally experience during their life cycle.

We were able to obtain measurements for daily temperature and precipitation from our field site, and it appears that both of these factors are significantly correlated with gene expression patterns. Precipitation was correlated with global gene expression patterns across the full development of the plant, while temperature was correlated with expression only in pre-bolting plants. This is unlikely to provide the full picture of responses to environmental conditions in the field, since for some genes, expression is probably associated with (i) complex, nonlinear responses to these environments, (ii) interactions between environmental signals, and (iii) responses to unmeasured environmental conditions. Future studies should provide greater breadth and resolution in environmental measurements. As more data becomes available, more complex relationships between gene expression and environmental features, including complex interactions between different variables, can be explored.

Despite the limitations of our environmental analyses, they confirm the role of several genes and gene sets to field environmental fluctuations, including *ARP6* [68], [90] and *HSP70* [71] to temperature, and *ROP10* [73] and *AAO3* [75] to precipitation. We did examine whether gene function for some of these loci showed fitness effects under variable or stressed environments (see Text S1, Figure S4 and Table S6). Using T-DNA insertion mutants for 14 genes that are associated with PC1^veg^ and PC2^veg^, we compared fruit number in mutant vs. wild-type lines under fluctuating temperature or decreased water environments. We only saw a significant environmentally-dependent effect of accession under changes in water availability, associated with the genes *AA03* and *ALDH7B4* [93]. Contrary to expectations, however, the decrease in fruit numbers in mutant vs. wild-type lines was observed in benign (and not stressful) environments (see Figure S4 and Table S6). More intensive studies with a larger sample of genes may yet reveal fitness consequences of other ecologically-relevant genes identified in our analysis.

While there have been tremendous strides in understanding the molecular genetic networks underlying plant phenotypes, we still know very little about what happens in natural wild environments. Indeed, there is growing interest in the study of the genetics of adaptation in ecological field environments, especially as related to climatic variables [94], [95]. As we begin to study the genetic networks associated with plant environmental responses [91], [92], we can correlate molecular networks with gene expression profiles in the wild, identify natural variation in genes and genetic networks and associated microevolutionary adaptations, and establish relationships between gene functions and organismal phenotypes [6]. This will allow us to link gene functions to ecologically relevant responses of plants to their lives in the wild, providing the foundation for the study of ecological genomics and ecological systems biology that can illuminate the molecular basis of species adaptations to real-world environments.

## Materials and Methods

### Study material and sites

We chose Bay-0 and Sha because they are rapid-cycling spring annuals that germinate and flower under Northeastern US field conditions [93]. These accessions have been genotyped at >1,000 gene fragments and thus have a large amount of SNP markers available for further genetic characterization [45], and are the progenitors of a recombinant inbred mapping population with over 165 core lines that can be used for future QTL mapping studies [44]. The field site at the Cold Spring Harbor Laboratory greenhouses has similar, but milder climatic conditions to Bristol, Rhode Island, which is the site of previous *A. thaliana* field projects [25], [93]–[95], and we had previously grown Bay-0 and Sha accessions in this field site in the fall/winter/spring of 2006–2007.

### Sampling and microarray experiments

We stratified seeds of Bay-0 and Sha for four days at 4°C and planted them in flats in a mixture of equal parts topsoil, sand and peat moss. We left the seeds under domes to germinate in an ambient temperature greenhouse for 5 days, and moved them outside on day 10 to acclimate the plants before transplanting to the field. On day 13 (27 April 2008 at the ∼4-leaf stage), we planted seedlings in a 2-m^2^ field grid marked off every 2 cm^2^, and distributed the two accessions across the grid in a completely randomized design. For each accession, we collected three replicates of the entire shoot of two individuals every three days, between 4.5–5.0 hours after sunrise, starting five days after transplant until bolting was observed for each accession (see Figure 1A). We collected replicates for each sample within a 15–20 minute window in a given day. We recorded bolting day as the day when at least 50% of the plants of an accession had initiated bolting. On this day for each accession (sample 6 for Bay-0 and sample 8 for Sha), we collected three sets of replicates from both bolted and non-bolted individuals. Three days after this bolting date for each accession, we collected the final sample of bolted shoots. Bolting occurred at 34 days for Bay-0, and we collected six vegetative and two bolting timepoints. The Sha accession bolted at 40 days after planting, and we collected eight vegetative and two bolting timepoints.

We collected a total of 18 samples in triplicate except for in Sha, where one replicate was lost in the sixth sample timepoint. For each replicate of each sample, we extracted total RNA using the RNAEasy plant mini kit (QIAGEN), using all of the above ground tissue for both plants. In the later stages of development, the total material for reach replicate exceeded the limits recommended for the Qiagen spin columns. In those cases, we used twice as much RLT buffer to suspend the frozen finely ground tissue and transferred half of the lysate to each of two spin columns. The two halves of the sample were kept separate through the collection, but were combined before quantification and hybridization to the microarray chips. The New York University Medical Center Genome Core Facility performed the hybridization of RNA and scanning to Affymetrix ATH1 chips according to manufacturer's protocols (Affymetrix).

### Data analysis

In order to compare our results to those observed in the ATGenExpress [29] data set, we used the Affymetrix Microarray Suite 5 (MAS 5) algorithm [47], [96] to determine if genes were expressed at any time point. Genes were considered expressed if they were observed in all three replicates of a sample. We used JMP/Genomics with the SAS statistical package (Version 9.1.3 for Windows; SAS Institute, Cary, NC, USA) and Virtual Plant 1.0 [65] for all initial PVCA, PCA, correlations, ANOVA and Gene Ontology (GO) analyses. Because the ATH1 microarray was designed based on the Col-0 accession, different single feature polymorphisms (SFPs) for probes within each probe set may exist for Bay-0 and for Sha, and probe mis-hybridization may occur when examining the transcriptome of Bay-0 and Sha [97]. To correct for this problem, we ran analyses on different imports of the raw (.cel file) data filtering the appropriate probes that contained SFPs for Bay-0, Sha, or for both depending on the analysis. After importing the raw data into JMP/Genomics with the appropriate filter, we background transformed the data with RMA across the collection of microarrays. Raw expression was summarized by probeset with a median polish and log_2_ transformation. We used the TAIR 9 annotation file to obtain the probe to gene matches.

We used principal variance components analyses [PVCA; 54] to examine global expression trends associated with accession and flowering status. The PVCA approach first reduces the dimensionality of the data set with PCA, and then computes variance components by fitting a mixed linear model to each principal component, treating each factor of interest in the model as a random effect (including continuous variables). We used the model PC*i* = accession+flowering status+accession-by-flowering status+error, where *i* indicates each principal component, starting with 1 and continuing through all principal components calculated in the PCA. The variance component for each factor is obtained by a weighted averaging across the values calculated for each principal component, weighted by the eigenvalues for the corresponding principal component. We used the same factors in a mixed model ANOVA to directly fit the model to gene expression (ANOVA model: gene expression = accession+flowering status+accession-by-flowering status+error). We also used the same PVCA and mixed model ANOVA approach to examine a larger model that incorporated age and the environmental factors maximum daily temperature, minimum daily temperature and daily precipitation (ANOVA model: PC*i* or gene expression = age+flowering status+minimum temperature+maximum temperature+precipitation+error) within each accession.

Finally, we used the EDGE program designed for significance testing of time course microarray data that fits a cubic spline to gene expression levels across time and tests for significant deviation from an invariant gene expression pattern [57], [58]. We ran a GO analysis in JMP Genomics to identify association with gene ontology categories for each cluster.

### Cluster analysis

We used K-means clustering on the mean expression values of the significant genes from a stringent pairwise ANOVA analysis (FDR q<0.01) for Bay-0 (3,827 genes) and Sha (8,215 genes). Although an r = 0.7 has been arbitrarily used in other microarray analyses to define the number of clusters within a data set, we used the silhouette function in MATLAB (MathWorks 2009) to find an appropriate number of distinct clusters of genes that behave similarly across the data sets [59]. The silhouette statistic is a pairwise method of evaluating the amount of similarity of individuals within a cluster compared to each of the individuals within each of the other clusters. By running this statistic on an increasing number of clusters, the silhouette approach allowed us to identify an appropriate number of clusters given the structure of the data [98]. We identified the appropriate number of distinct clusters within each accession when the average silhouette value of the worst cluster became 0 and each successive increase in the number of clusters continued to show that the average silhouette value of the worst cluster was 0 or less than zero. We also ran GO enrichment analysis to identify association with gene ontologies for each cluster using JMP Genomics. To identify which clusters are associated with age, flowering status or environmental factors, we ran PVCA on all of the genes in each cluster separately for all Bay-0 and Sha clusters.

### Stress annotation analysis

We created a functional annotation file based on previously published microarray data that had reported differential regulation of specific genes in response to several abiotic and biotic stresses. These include 118 high light response genes [60]; 4,972 cold, 1,562 drought, 3,990 heat, 5,842 osmotic, 511 oxidative, 5,148 salt, 1,219 toxins, 3,792 UVB and 1,771 wounding response genes [61]. Biotic stresses include 97 [62] and 3,687 [63] RNA virus response genes; 2,034 bacterial pathogen response genes, 151 fungal pathogen response genes, 2,397 herbivore response genes [64]. Combining these data sets, more than half of the genes on the ATH1 array were associated with at least one stress (13,153 of 22,800 genes).

### Principal component analyses of vegetative stages

We performed a more detailed principal component analysis (PCA) in vegetative stages using JMP Genomics (SAS). Probes were normalized and centered to zero to determine the main trends of the variation across the samples. PCA was done using only genes that are significantly expressed (q<0.05) in both Bay-0 and Sha in vegetative stages (8,954 genes, representing 40% of the original dataset). Thus, trends in gene expression in each sample can be represented as PC loadings (i.e., PC1^veg^, PC2^veg^, PC3^veg^) in an allometric gene expression scale. To assess whether trends in gene expression were influenced by environmental factors or development, we ran a multivariate regression analysis using the PC^veg^ scores as a response variable to maximum daily temperature (TMAX), daily precipitation (PPT), rosette leaf number (RLN), rosette diameter (RD), and plant age in the following model [PC^veg^ = TMAX+PPT+RLN+RD+age+error]. Variance inflation factors were below 10, indicating low co-linearity between variables. We ran the regression analysis on the first five PC axis scores using an FDR of 0.05.

To identify genes contributing to each PC axis, we selected genes that showed extreme PC loadings for each of the PC^veg^ from the upper and lower 2.5% of the quantile distributions in the PC loadings, which identified a subset of 448 genes in each PC^veg^. Because the PCs are orthogonal axes, the genes driving the variation in PC1^veg^ do not intersect with the genes in PC2^veg^ and PC3^veg^. The gene lists were analyzed for GO enrichment (p≤0.01) to identify significantly over-represented functional gene classes in Virtual Plant 1.0 [65].

Several genes in these gene lists caught our attention, including *ARP6* and *ROP10*. To obtain lists of genes that are co-expressed with *ARP6* and *ROP10*, Pavlidis Template Matching was done using the Multiple Array Viewer software, with absolute correlation coefficients as thresholds [72].

## Supporting Information

Figure S1 Replicate quality for eight Bay-0 and 10 Sha samples. Average pairwise correlations between triplicate samples (with the exception of Sha timepoint 7 for which one sample was lost) for each sample.
(TIF)

Figure S2 Number of genes considered “present” in all three replicates by Affymetrix MAS 5 algorithm. Shown are counts of genes present for each sample for Bay-0 and Sha as well as across all Bay-0 and across all Sha and across both. Also shown are the total gene counts with “present” calls across all three replicates in the whole ATGen Express data set and what we consider the relevant comparison of non-mutant, above ground, non-senescent tissue.
(TIF)

Figure S3 Gene Ontology (GO) categories for genes that were considered “absent” in all three replicates of at least one sample. Approximately 1/3 of the genome of both Bay-0 (8,322 genes) and Sha (7,948) were not detected in this study: (A) By far the majority in both accessions were unannotated genes. (B) Typically the number of genes in each GO category was similar across the two accessions.
(TIF)

Figure S4 Fitness assays showing the mutants that had significant accession-by-environment interaction as response to drought.
(TIF)

Table S1 GO categories for genes that were detected as significantly different in an ANOVA for the main effects of accession and flowering status. Although there were 306 genes that showed a significant interaction.
(DOCX)

Table S2 List of genes most strongly correlated to PC1^veg^ (upper and lower most 2.5% of the quantile distributions).
(DOCX)

Table S3 List of genes correlated to PC1^veg^ (upper and lower 2.5% to 5% of the quantile distributions).
(DOCX)

Table S4 List of genes most strongly correlated to PC2^veg^ (upper and lower most 2.5% of the quantile distributions).
(DOCX)

Table S5 List of genes correlated to PC2^veg^ (upper and lower 2.5 to 5% of the quantile distributions).
(DOCX)

Table S6 Significant differences in fitness (fruit number) associated with gene functions in fluctuating temperature (PC1^veg^) and water availability (PC2^veg^) treatments
(DOCX)

Text S1 Fitness analysis.
(DOC)
