# Supplementary figures and images for: Genome-Wide Patterns of Arabidopsis Gene Expression in Nature
Source: PLoS Genet. 2012 Apr 19;8(4):e1002662. doi: 10.1371/journal.pgen.1002662 (PMC3330097; doi:10.1371/journal.pgen.1002662)

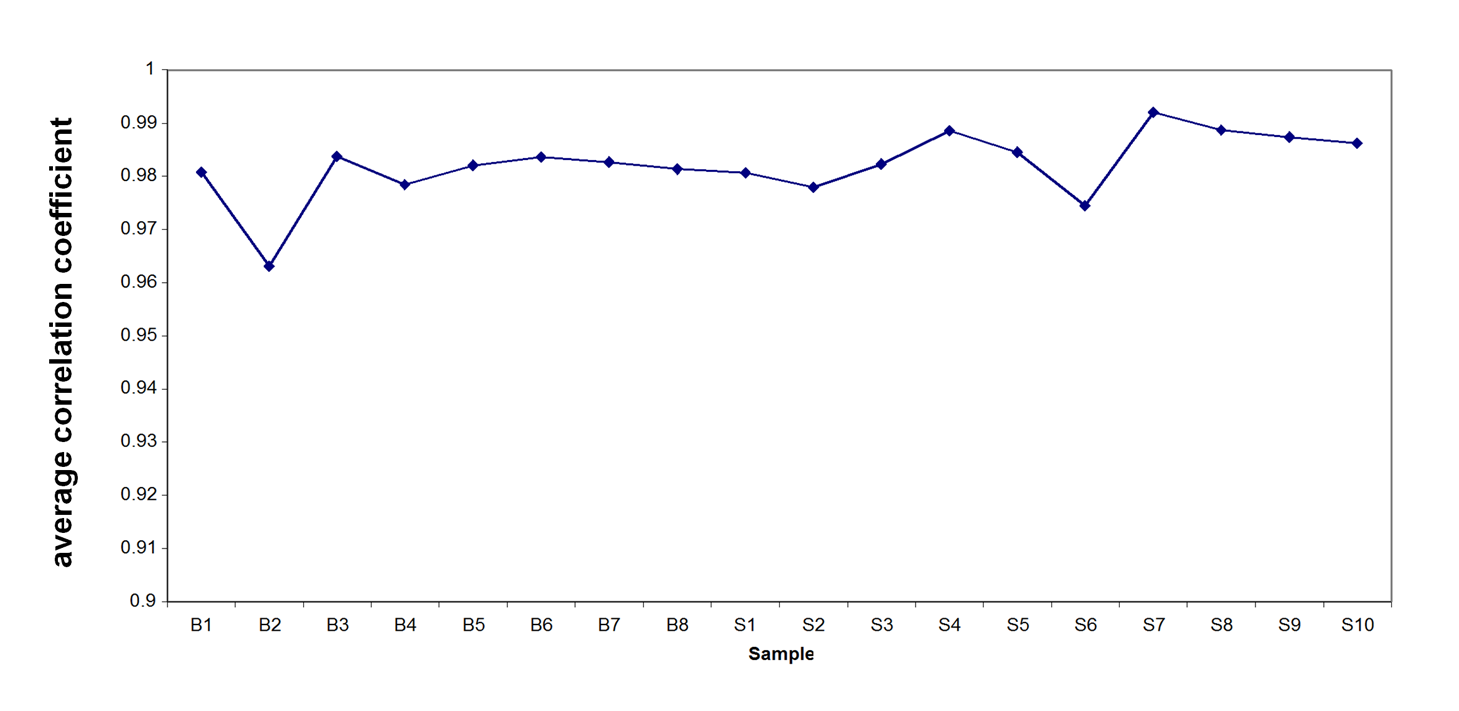

Supplement: Figure S1 — Replicate quality for eight Bay-0 and 10 Sha samples. Average pairwise correlations between triplicate samples (with the exception of Sha timepoint 7 for which one sample was lost) for each sample. (TIF) [file pgen.1002662.s001.tif]

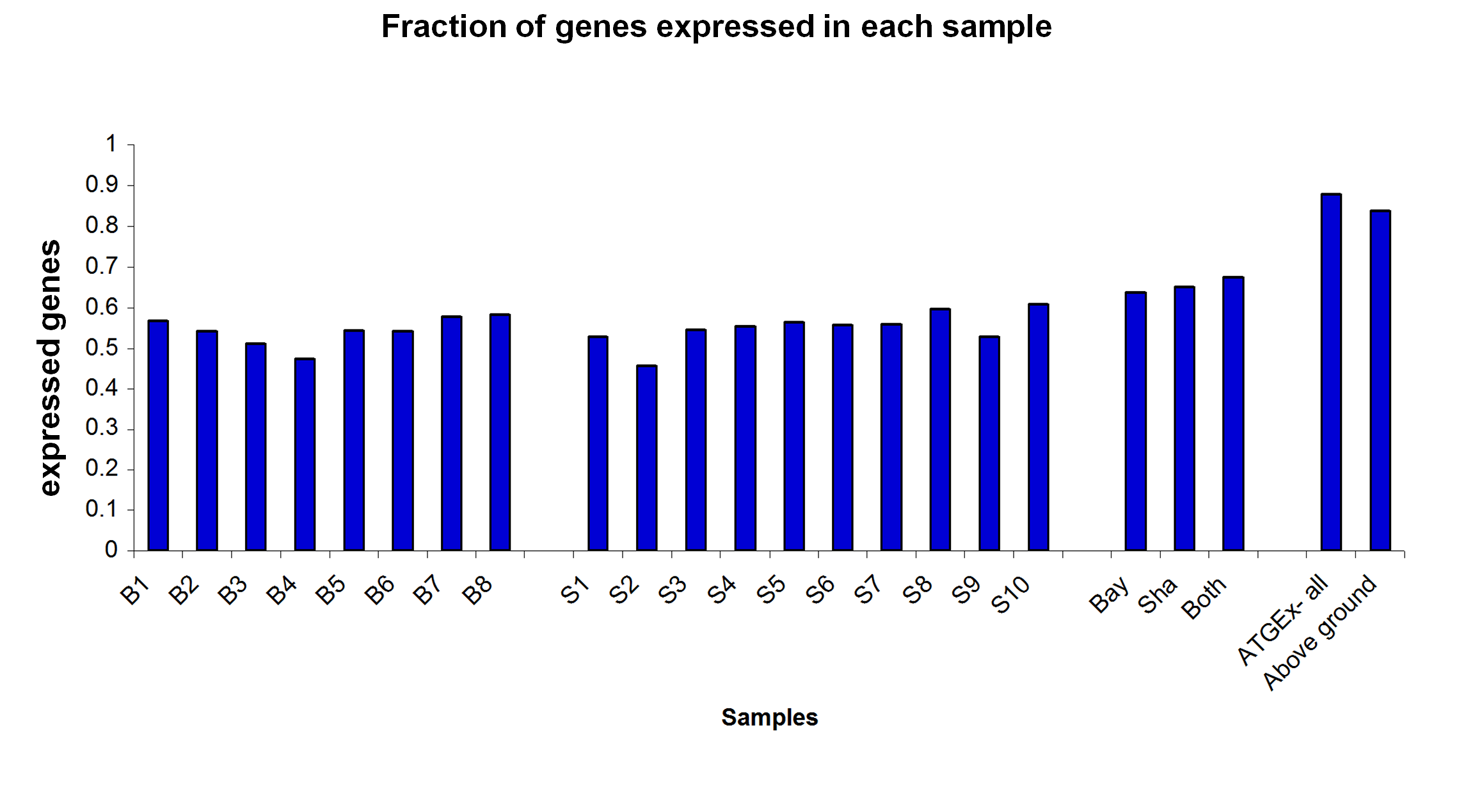

Supplement: Figure S2 — Number of genes considered “present” in all three replicates by Affymetrix MAS 5 algorithm. Shown are counts of genes present for each sample for Bay-0 and Sha as well as across all Bay-0 and across all Sha and across both. Also shown are the total gene counts with “present” calls across all three replicates in the whole ATGen Express data set and what we consider the relevant comparison of non-mutant, above ground, non-senescent tissue. (TIF) [file pgen.1002662.s002.tif]

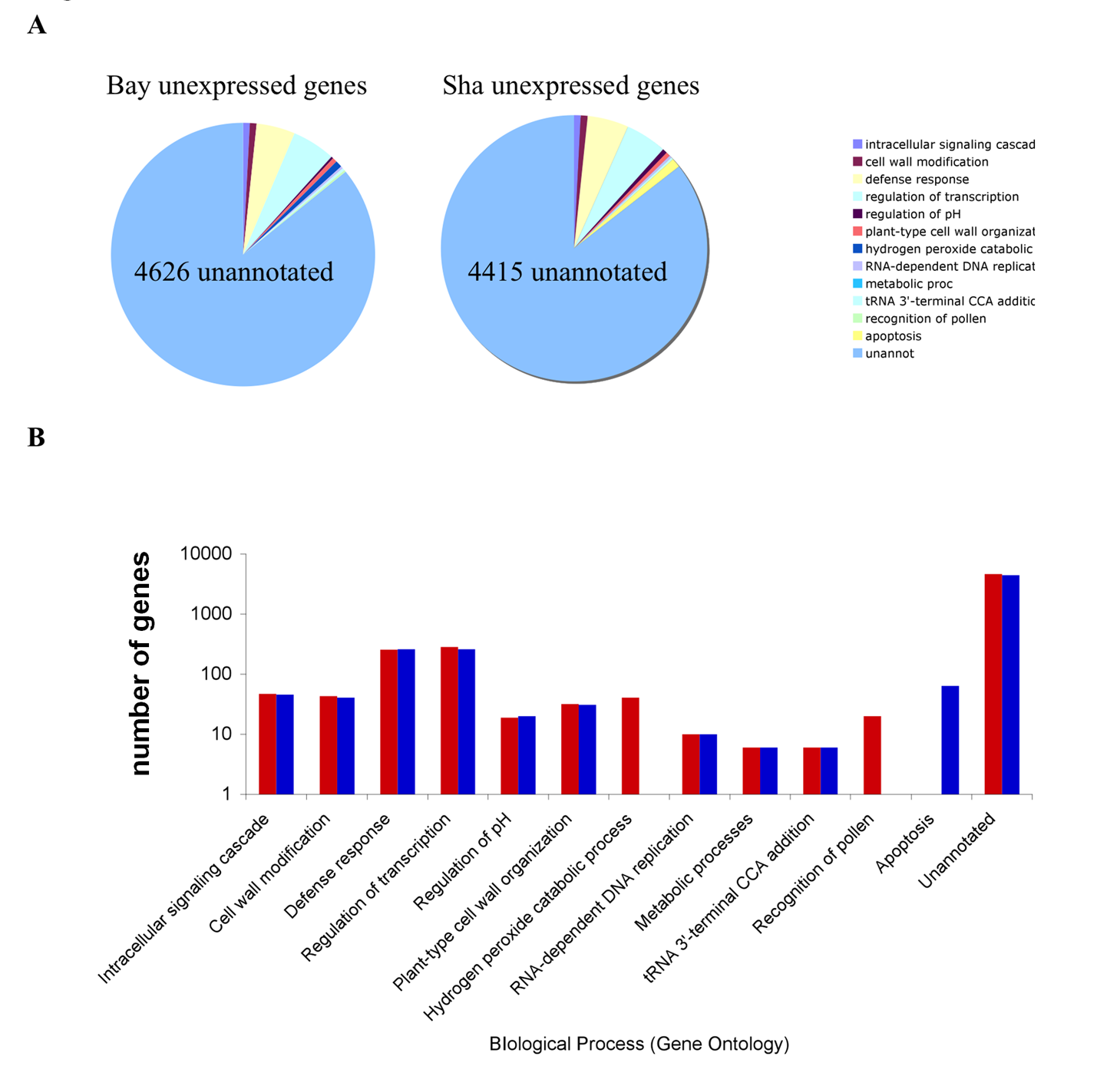

Supplement: Figure S3 — Gene Ontology (GO) categories for genes that were considered “absent” in all three replicates of at least one sample. Approximately 1/3 of the genome of both Bay-0 (8,322 genes) and Sha (7,948) were not detected in this study: (A) By far the majority in both accessions were unannotated genes. (B) Typically the number of genes in each GO category was similar across the two accessions. (TIF) [file pgen.1002662.s003.tif]

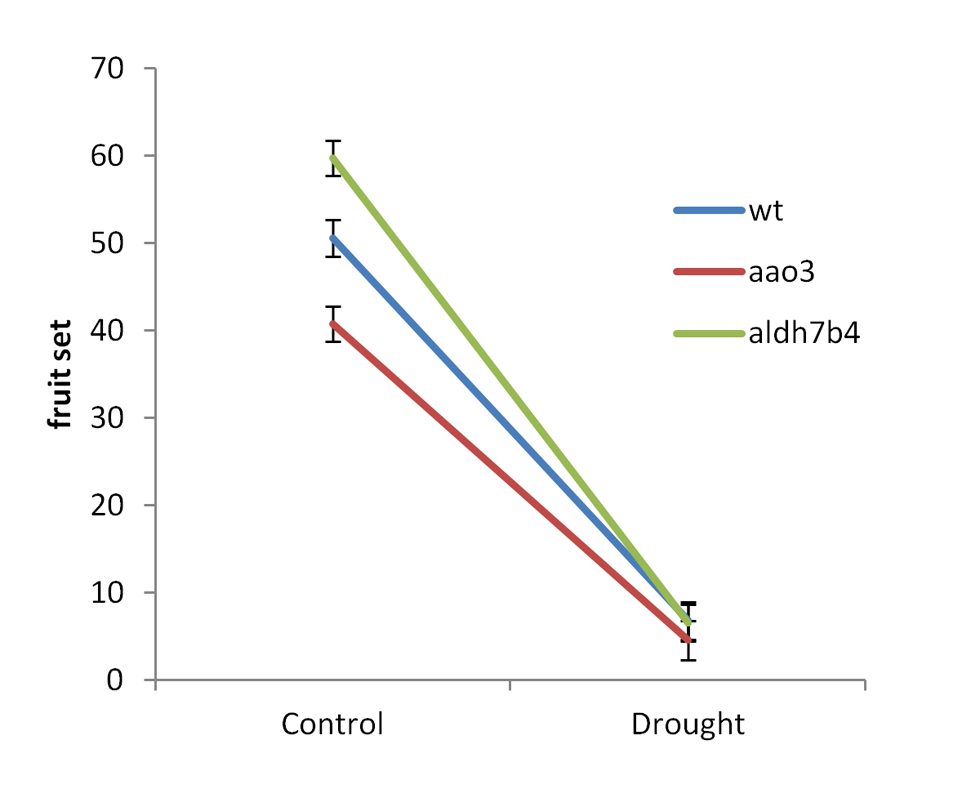

Supplement: Figure S4 — Fitness assays showing the mutants that had significant accession-by-environment interaction as response to drought. (TIF) [file pgen.1002662.s004.tif]
