# Supplementary material for: Genome-Wide Patterns of Arabidopsis Gene Expression in Nature
Source: PLoS Genet. 2012 Apr 19;8(4):e1002662. doi: 10.1371/journal.pgen.1002662 (PMC3330097; doi:10.1371/journal.pgen.1002662)
Supplement: Table S1 — GO categories for genes that were detected as significantly different in an ANOVA for the main effects of accession and flowering status. Although there were 306 genes that showed a significant interaction. (DOCX) [file pgen.1002662.s005.docx]

**Supplemental Table S1.** GO categories for genes that were detected as significantly different in an ANOVA for the main effects of accession and flowering status. Although there were 306 genes that showed a significant interaction between these two terms, none of the GO categories were over- (or under-) represented.
