# Supplementary material for: Genome-Wide Patterns of Arabidopsis Gene Expression in Nature
Source: PLoS Genet. 2012 Apr 19;8(4):e1002662. doi: 10.1371/journal.pgen.1002662 (PMC3330097; doi:10.1371/journal.pgen.1002662)
Supplement: Table S2 — List of genes most strongly correlated to PC1veg (upper and lower most 2.5% of the quantile distributions). (DOCX) [file pgen.1002662.s006.docx]

**Table S2.** List of genes most strongly correlated to PC1^veg^ (upper and lower most 2.5% of the quantile distributions).

AT2G16890 AT1G01770 AT1G69870 AT1G69370 AT3G24120 AT3G43980 AT2G35190 AT4G14230 AT1G14820 AT1G09010 AT1G03630 AT5G37600 AT4G34950 AT3G50970 AT2G36000 AT1G13100 AT2G29020 AT5G35980 AT5G04980 AT3G60610 AT4G23570 AT4G14465 AT1G48650 AT1G01420 AT1G71990 AT5G08470 AT2G30530 AT5G60870 AT3G29350 AT1G21540 AT4G12400 AT2G32180 AT4G09670 AT5G14450 AT1G51400 AT1G07420 AT5G67290 AT5G57000 AT5G56940 AT2G35780 AT1G27410 AT3G44010 AT2G30800 AT1G70210 AT5G50800 AT1G10240 AT5G16290 AT5G03720 AT5G43860 AT1G32640 AT5G53170 AT2G25930 AT1G16780 AT1G22770 AT2G02080 AT3G13330 AT1G07480 AT3G50900 AT5G26210 AT4G04450 AT3G18760 AT5G06340 AT4G35850 AT3G33530 AT3G15010 AT4G14930 AT1G66080 AT5G38220 AT5G24460 AT5G42310 AT1G24530 AT3G25230 AT3G18710 AT5G58380 AT1G21410 AT5G25130 AT3G13470 AT3G08010 AT3G51010 AT4G22756 AT1G64200 AT2G29570 AT5G47650 AT2G23080 AT5G52310 AT5G28840 AT5G35220 AT2G47890 AT1G61065 AT4G11330 AT1G04850 AT5G52860 AT5G14680 AT2G32650 AT5G66200 AT4G28270 AT4G02680 AT3G23820 AT4G18210 AT3G62260 AT5G56190 AT5G11150 AT3G51450 AT3G15030 AT1G75900 AT1G20840 AT4G25420 AT2G36460 AT2G20790 AT5G27400 AT3G62330 AT5G35530 AT3G23280 AT2G29090 AT1G14980 AT5G21010 AT4G14900 AT2G37180 AT2G28840 AT1G13110 AT5G63320 AT4G26790 AT4G25960 AT3G11290 AT5G43260 AT5G05730 AT5G53140 AT3G02720 AT2G29630 AT1G29720 AT5G59910 AT4G21180 AT2G44760 AT2G37478 AT1G55810 AT4G34030 AT3G56750 AT5G60840 AT4G38130 AT3G22880 AT2G31890 AT2G41880 AT1G10960 AT2G37170 AT1G62810 AT3G15410 AT2G27600 AT1G69680 AT3G06950 AT2G21660 AT5G62650 AT4G38620 AT1G23740 AT3G52310 AT5G03700 AT4G40060 AT4G17090 AT1G54050 AT1G78800 AT3G15260 AT4G11660 AT3G28270 AT3G48460 AT1G07350 AT1G22885 AT3G02250 AT4G24470 AT2G19720 AT5G56600 AT3G53500 AT3G54480 AT3G52610 AT2G22125 AT1G19860 AT1G71040 AT1G77370 AT5G14940 AT3G58050 AT5G47420 AT5G64510 AT5G18100 AT3G60320 AT1G03860 AT5G24500 AT3G19590 AT2G04350 AT3G09760 AT1G16510 AT3G63480 AT2G22090 AT3G10985 AT1G20696 AT5G13930 AT1G52730 AT1G57600 AT3G19000 AT1G53570 AT5G08100 AT2G46610 AT4G18220 AT1G62390 AT5G50370 AT2G42450 AT5G65490 AT3G29590 AT1G73650 AT2G36470 AT3G04760 AT5G35210 AT1G12910 AT3G24590 AT1G21670 AT5G01650 AT2G45160 AT3G14690 AT2G29210 AT2G31380 AT3G60250 AT1G07280 AT5G09870 AT1G55520 AT1G48830 AT4G35510 AT2G28550 AT5G51570 AT3G53670 AT1G07360 AT3G45260 AT2G02350 AT2G02100 AT3G04605 AT5G53000 AT1G80780 AT1G51940 AT1G75130 AT3G19930 AT3G09660 AT1G52200 AT2G20560 AT1G23860 AT2G25570 AT5G65260 AT3G06850 AT1G72800 AT3G13290 AT3G22440 AT4G36960 AT1G55170 AT4G26700 AT1G18460 AT2G11810 AT5G23520 AT1G16350 AT1G78570 AT2G33740 AT2G15890 AT3G12050 AT3G46000 AT4G29330 AT3G51180 AT3G25805 AT5G05170 AT3G08510 AT5G51660 AT4G14605 AT3G21560 AT3G29230 AT1G71880 AT2G31070 AT1G01060 AT3G26450 AT5G03290 AT4G33950 AT1G53560 AT2G33735 AT1G09980 AT5G58590 AT1G09350 AT5G05540 AT5G56280 AT3G14990 AT4G02150 AT3G03630 AT1G30260 AT2G39900 AT1G67850 AT3G08030 AT1G21120 AT5G42520 AT2G31190 AT3G22220 AT5G12020 AT2G39710 AT5G39040 AT4G20870 AT4G38960 AT5G61520 AT2G37480 AT5G27710 AT1G20693 AT5G14760 AT3G24170 AT1G25560 AT4G15560 AT5G46450 AT5G67150 AT3G50070 AT5G48030 AT1G21560 AT1G30070 AT5G57100 AT2G28720 AT1G51805 AT2G44640 AT1G47530 AT5G06280 AT1G07470 AT2G30320 AT4G37560 AT1G23180 AT5G06050 AT5G61670 AT2G36830 AT5G52120 AT1G18680 AT5G55610 AT4G24830 AT2G36970 AT5G12110 AT5G56850 AT4G38580 AT2G17470 AT3G53668 AT1G77180 AT2G38820 AT3G26520 AT1G16570 AT1G14580 AT3G13300 AT2G39450 AT4G38420 AT2G44660 AT4G15430 AT1G78660 AT3G14790 AT5G52640 AT2G44730 AT1G78060 AT1G77850 AT1G24330 AT1G22740 AT2G31360 AT5G46700 AT3G50000 AT1G54390 AT1G29260 AT3G06500 AT3G27300 AT3G57780 AT1G50110 AT5G47830 AT5G26570 AT4G25500 AT5G03440 AT1G55310 AT1G18660 AT2G37580 AT3G18210 AT3G24500 AT3G56590 AT2G41700 AT3G55520 AT5G49330 AT2G15970 AT3G16050 AT4G32690 AT1G22070 AT1G14400 AT3G16910 AT5G51440 AT3G62010 AT2G47490 AT1G67080 AT3G09540 AT1G09240 AT3G11040 AT5G05670 AT1G20540 AT3G47560 AT4G24510 AT5G58900 AT1G71030 AT4G17550 AT1G60170 AT1G32130 AT2G28930 AT5G08640 AT3G49500 AT3G52720 AT5G62930 AT3G02990 AT1G03905 AT5G42420 AT4G30490 AT3G50700 AT4G01330 AT1G02970 AT2G44670 AT4G38020 AT2G19860 AT4G30650 AT4G32060 AT2G37040 AT2G29450 AT1G25520 AT4G29070 AT5G60890 AT3G57810 AT2G34620 AT1G05805 AT1G50010 AT1G74310 AT5G47880 AT2G17900 AT1G74530 AT1G74430 AT3G12580 AT4G21610 AT2G02070 AT5G13720 AT4G18390 AT4G38490 AT5G44870 AT2G23910 AT3G08970 AT5G01770 AT4G28550 AT4G24040 AT5G06530 AT5G39790 AT5G56180 AT1G76080 AT5G58770 AT1G58350 AT3G15430 AT2G26070 AT2G37790 AT5G24470 AT1G09140 AT5G16140 AT1G13930 AT1G26740 AT2G21520 AT1G26260 AT3G01150 AT5G08270 AT3G63260 AT5G07580
