# Supplementary material for: Genome-Wide Patterns of Arabidopsis Gene Expression in Nature
Source: PLoS Genet. 2012 Apr 19;8(4):e1002662. doi: 10.1371/journal.pgen.1002662 (PMC3330097; doi:10.1371/journal.pgen.1002662)
Supplement: Table S3 — List of genes correlated to PC1veg (upper and lower 2.5% to 5% of the quantile distributions). (DOCX) [file pgen.1002662.s007.docx]

**Table S3.** List of genes correlated to PC1^veg^ (upper and lower 2.5% to 5% of the quantile distributions).

AT5G15970 AT1G12240 AT1G16500 AT1G55880 AT5G20110 AT3G57690 AT2G25730 AT1G04820 AT5G07990 AT1G28530 AT4G35040 AT4G39570 AT5G56790 AT4G19390 AT4G23410 AT4G30450 AT4G09020 AT5G27730 AT3G04230 AT5G20400 AT2G34960 AT3G27020 AT2G46510 AT2G27040 AT5G49390 AT2G38160 AT3G18430 AT1G53230 AT1G54650 AT3G28700 AT1G49230 AT3G01370 AT1G64850 AT4G20760 AT1G73730 AT3G54140 AT5G01880 AT3G16630 AT1G71750 AT5G13090 AT2G20480 AT5G11340 AT5G37670 AT2G07690 AT1G32400 AT5G64740 AT3G17611 AT5G16340 AT4G30330 AT3G23990 AT1G07140 AT5G21170 AT5G22850 AT3G55580 AT5G03520 AT2G35700 AT2G46580 AT4G33630 AT5G62575 AT4G15415 AT1G70270 AT5G50280 AT5G62700 AT3G15760 AT2G17730 AT1G51440 AT1G14140 AT2G22450 AT2G32920 AT4G04860 AT5G62690 AT1G11330 AT4G39090 AT5G05270 AT1G67750 AT3G25110 AT4G16950 AT3G09700 AT5G27700 AT1G22610 AT1G67800 AT5G66750 AT5G13440 AT5G15090 AT5G42950 AT1G17110 AT2G35605 AT1G01500 AT5G43880 AT5G62980 AT5G15960 AT5G09320 AT2G44050 AT5G41600 AT5G09860 AT5G54800 AT3G59100 AT4G23600 AT3G09210 AT3G05640 AT4G30660 AT1G80850 AT2G16500 AT4G17540 AT1G22040 AT1G54410 AT4G25450 AT4G08850 AT3G50880 AT5G64080 AT2G38750 AT1G52630 AT1G62430 AT5G06980 AT5G04280 AT4G30550 AT2G21880 AT2G42710 AT1G49170 AT2G38465 AT5G20660 AT2G46830 AT5G58110 AT3G60350 AT5G62630 AT5G53150 AT5G17220 AT1G27930 AT4G04750 AT5G42730 AT5G51710 AT2G18400 AT2G33620 AT3G03450 AT1G19370 AT5G13430 AT1G69950 AT1G74800 AT5G52450 AT3G47630 AT5G44785 AT4G39040 AT1G53710 AT5G61880 AT1G75190 AT5G28750 AT5G53200 AT5G12030 AT5G01090 AT1G10050 AT1G16870 AT5G10630 AT2G37020 AT1G72500 AT1G03420 AT1G19740 AT4G32950 AT3G57520 AT3G25040 AT1G14360 AT3G05800 AT2G48120 AT1G72090 AT1G72510 AT2G47850 AT1G53035 AT3G57800 AT1G31420 AT3G17470 AT4G25480 AT5G50200 AT5G23860 AT2G35840 AT5G17230 AT1G02180 AT1G20980 AT3G12570 AT2G44490 AT5G50930 AT3G10410 AT2G20940 AT3G07770 AT2G33255 AT5G61590 AT5G25450 AT5G46270 AT4G00730 AT4G33670 AT3G06483 AT3G16230 AT3G22840 AT3G55120 AT2G03390 AT3G21200 AT5G50400 AT1G75330 AT3G27060 AT5G11600 AT4G26130 AT3G15840 AT4G24480 AT2G09970 AT3G60340 AT4G38660 AT5G59030 AT3G18640 AT3G52660 AT2G30350 AT5G28390 AT5G59400 AT4G12640 AT1G06000 AT1G78020 AT3G13845 AT2G40935 AT5G19030 AT1G53650 AT2G26150 AT2G38760 AT3G21790 AT3G43720 AT5G52190 AT2G43630 AT1G49400 AT3G48000 AT2G24150 AT3G29810 AT5G56350 AT5G51840 AT1G12470 AT1G49720 AT1G52230 AT1G70630 AT5G41880 AT1G46768 AT3G04710 AT2G33380 AT1G22190 AT3G44450 AT1G12850 AT2G38290 AT4G12120 AT2G02170 AT3G18270 AT1G68050 AT2G38180 AT3G24190 AT2G16365 AT4G27440 AT2G29980 AT5G37780 AT1G14900 AT1G23140 AT4G10120 AT3G33520 AT5G15640 AT1G54130 AT3G61550 AT2G24220 AT1G53510 AT4G14690 AT3G47500 AT4G01510 AT2G07050 AT2G04030 AT5G25110 AT5G17030 AT5G49010 AT3G10150 AT2G43850 AT4G34215 AT2G37260 AT5G61920 AT3G09350 AT1G04620 AT3G07090 AT5G65890 AT1G65960 AT5G60710 AT5G09740 AT1G72170 AT3G22120 AT1G26110 AT4G33000 AT5G56520 AT3G03380 AT1G53730 AT1G78680 AT1G10760 AT1G52340 AT5G14550 AT1G74100 AT5G03470 AT5G25210 AT5G66880 AT4G14870 AT5G50840 AT3G26510 AT5G11950 AT3G02180 AT4G29190 AT3G53740 AT2G34640 AT5G26600 AT5G05680 AT1G51060 AT4G33660 AT5G35700 AT3G62000 AT5G07842 AT5G42800 AT5G56140 AT1G15950 AT4G11600 AT5G08570 AT5G15050 AT5G06570 AT5G52820 AT1G52720 AT3G12490 AT2G29390 AT1G21060 AT2G25790 AT2G33440 AT4G24730 AT4G03020 AT1G28600 AT1G69570 AT5G07840 AT5G45410 AT2G41160 AT1G62710 AT2G40280 AT1G10360 AT4G00710 AT2G03800 AT5G16370 AT4G14840 AT3G58010 AT1G71696 AT2G26440 AT1G26460 AT4G02980 AT3G44160 AT2G30980 AT5G23720 AT1G34190 AT4G18810 AT3G06320 AT4G13720 AT1G18650 AT4G22670 AT4G24280 AT3G62240 AT2G41460 AT1G62480 AT1G48210 AT1G07170 AT1G07980 AT1G21190 AT1G79510 AT2G28000 AT4G25030 AT2G27720 AT5G13960 AT2G28320 AT2G27580 AT3G02520 AT3G54610 AT2G21560 AT3G51520 AT4G35890 AT3G46830 AT2G32120 AT3G12200 AT3G15160 AT2G36290 AT2G35010 AT4G34890 AT4G35030 AT3G13570 AT5G12080 AT5G15860 AT3G14180 AT1G30210 AT1G57870 AT5G17190 AT5G02190 AT5G48570 AT1G45000 AT5G62020 AT2G21620 AT5G10870 AT5G18790 AT2G01930 AT3G12530 AT1G48330 AT5G23410 AT2G37340 AT4G36920 AT3G51660 AT2G37500 AT3G05220 AT1G05210 AT1G71900 AT5G65040 AT3G48620 AT1G60960 AT1G01100 AT2G48100 AT5G13830 AT2G19450 AT4G24690 AT2G47580 AT2G19460 AT5G20040 AT4G30530 AT1G03610 AT5G13400 AT5G37260 AT4G34900 AT5G10460 AT4G23290 AT5G12310 AT5G27760 AT5G55120 AT5G04360 AT1G78490 AT1G66940 AT5G02530 AT5G18310 AT3G27390 AT2G25140 AT4G36440 AT5G65640
