# Supplementary material for: Genome-Wide Patterns of Arabidopsis Gene Expression in Nature
Source: PLoS Genet. 2012 Apr 19;8(4):e1002662. doi: 10.1371/journal.pgen.1002662 (PMC3330097; doi:10.1371/journal.pgen.1002662)
Supplement: Table S4 — List of genes most strongly correlated to PC2veg (upper and lower most 2.5% of the quantile distributions). (DOCX) [file pgen.1002662.s008.docx]

**Table S4.** List of genes most strongly correlated to PC2^veg^ (upper and lower most 2.5% of the quantile distributions).

AT3G62930 AT3G10080 AT3G29170 AT1G73980 AT1G64720 AT2G39550 AT1G02850 AT1G77690 AT1G08180 AT1G05385 AT5G37550 AT5G62200 AT3G07720 AT1G12250 AT5G38895 AT4G24220 AT1G10310 AT4G39540 AT5G10730 AT3G26440 AT1G80560 AT4G30610 AT4G15545 AT1G78380 AT1G20190 AT1G08550 AT1G53670 AT3G06860 AT2G41560 AT4G38680 AT2G35260 AT5G10650 AT4G32190 AT1G72680 AT3G13560 AT1G76570 AT1G20340 AT5G20910 AT5G05690 AT3G60630 AT1G36160 AT3G17000 AT5G60160 AT2G44750 AT4G34588 AT3G11270 AT5G47560 AT5G13980 AT1G11545 AT3G59700 AT5G64370 AT5G63880 AT1G12310 AT4G30110 AT4G38850 AT2G45180 AT5G64400 AT4G22240 AT4G19170 AT4G27570 AT3G09670 AT5G55580 AT5G59750 AT4G27700 AT1G62040 AT5G18670 AT5G60680 AT5G54870 AT4G35440 AT1G25230 AT4G31860 AT1G61800 AT1G63840 AT1G21600 AT1G08480 AT3G13910 AT1G50570 AT2G03140 AT1G55265 AT5G62610 AT3G47833 AT1G76930 AT2G16060 AT3G22845 AT1G17470 AT3G56940 AT1G02640 AT3G57630 AT1G54320 AT5G45820 AT4G11560 AT4G23820 AT1G11290 AT5G52410 AT4G38710 AT1G55960 AT1G02170 AT2G47450 AT4G02940 AT3G57040 AT2G16500 AT5G17860 AT3G08690 AT4G23050 AT1G74910 AT5G04750 AT3G21140 AT1G12920 AT1G09390 AT3G26820 AT5G67330 AT5G07290 AT4G24670 AT4G10100 AT1G17730 AT4G16146 AT1G80050 AT1G12990 AT1G07080 AT4G08180 AT1G26690 AT5G19540 AT3G24760 AT3G10420 AT1G19450 AT3G51440 AT2G40840 AT5G51545 AT1G02880 AT5G54960 AT5G22880 AT2G01290 AT3G55760 AT3G47850 AT5G48590 AT2G38120 AT3G55610 AT3G48040 AT4G14300 AT1G07320 AT1G33240 AT1G63540 AT4G02640 AT2G29420 AT2G27150 AT5G11420 AT3G59940 AT1G74670 AT1G07870 AT3G20320 AT5G65310 AT2G29460 AT5G25940 AT1G77770 AT5G62350 AT1G68520 AT5G02100 AT4G00040 AT2G37770 AT5G57170 AT2G33860 AT5G51110 AT3G01170 AT5G19820 AT5G17310 AT5G61290 AT2G18230 AT1G48320 AT4G32840 AT5G58640 AT1G49320 AT5G03650 AT1G70590 AT3G11230 AT3G51130 AT4G17840 AT2G45850 AT2G39250 AT3G57320 AT2G39800 AT1G32500 AT5G42810 AT1G13730 AT1G78140 AT3G04010 AT5G63220 AT4G02890 AT2G42610 AT3G17970 AT2G24060 AT1G63010 AT5G54130 AT3G62120 AT3G60420 AT3G26618 AT4G37760 AT3G48200 AT3G22210 AT2G46920 AT3G44880 AT3G05570 AT1G14685 AT1G77280 AT5G35790 AT3G46450 AT2G39725 AT1G49510 AT4G02340 AT3G17930 AT2G41680 AT1G49200 AT4G22570 AT2G29290 AT1G68560 AT1G78450 AT5G53280 AT5G62070 AT5G06960 AT4G39210 AT4G34830 AT5G12890 AT1G14720 AT1G55370 AT2G43760 AT2G39780 AT4G34590 AT1G72560 AT1G56430 AT5G27600 AT5G12390 AT2G01980 AT5G56090 AT5G61820 AT5G22920 AT2G39870 AT5G16800 AT5G67390 AT5G67420 AT5G01015 AT1G74850 AT3G63110 AT4G10060 AT5G62430 AT5G44190 AT1G47128 AT1G28670 AT5G53160 AT3G06420 AT4G03030 AT4G18930 AT4G33905 AT2G21270 AT1G02300 AT5G28770 AT3G50820 AT5G66910 AT1G75750 AT3G16240 AT5G19530 AT3G61960 AT1G28660 AT1G09900 AT2G40540 AT5G60620 AT5G06870 AT2G03750 AT4G27560 AT3G10520 AT1G69450 AT1G07110 AT4G17770 AT5G08330 AT1G70410 AT4G17460 AT3G02380 AT4G37190 AT2G31410 AT3G61580 AT5G04430 AT4G24660 AT1G54100 AT2G27420 AT5G54840 AT2G22310 AT1G01170 AT4G26060 AT2G31130 AT3G16370 AT2G22990 AT1G75180 AT1G03350 AT4G03140 AT5G40170 AT2G18300 AT1G36770 AT1G43130 AT4G27830 AT1G52080 AT4G38690 AT1G53800 AT1G30500 AT2G16740 AT1G50940 AT4G33490 AT1G79590 AT3G18050 AT4G30470 AT5G52210 AT3G03890 AT1G12500 AT1G21380 AT1G11580 AT4G02920 AT3G62880 AT2G29350 AT5G11840 AT4G19410 AT5G53860 AT4G04330 AT3G03310 AT1G65820 AT4G13195 AT4G25050 AT4G32250 AT2G39360 AT5G54680 AT3G12130 AT3G07470 AT1G77110 AT1G33560 AT3G18480 AT3G01550 AT1G77760 AT5G38110 AT3G06070 AT4G10970 AT3G58690 AT3G28050 AT2G46180 AT4G24160 AT2G45330 AT2G25850 AT3G23200 AT3G62190 AT5G22390 AT2G36990 AT2G23180 AT2G16700 AT1G74810 AT4G26530 ATCG01110 AT5G23090 AT1G06430 AT3G13740 AT3G51430 AT1G59700 AT1G22690 AT2G36835 AT5G64380 AT5G15830 AT2G46170 AT3G23560 AT1G36380 AT1G10210 AT4G34180 AT3G21630 AT4G38840 AT3G59660 AT5G38900 AT4G31170 AT3G13750 AT2G43510 AT4G22540 AT1G17700 AT5G55000 AT4G34740 AT4G28640 AT1G32450 AT5G13100 AT3G24460 AT2G21340 AT5G28300 AT3G26030 AT1G04220 AT3G61440 AT3G54380 AT5G51460 AT3G14340 AT4G04020 AT1G26220 AT2G25625 AT5G15350 AT3G56680 AT1G54340 AT1G80440 AT5G23600 AT5G40810 AT3G55800 AT5G57490 AT1G66150 AT1G45150 AT1G04300 AT1G65560 AT5G54080 AT1G65930 AT3G06080 AT1G25550 AT5G19430 AT2G37470 AT5G57060 AT3G63000 AT3G26840 AT3G19030 AT1G80900 AT3G28530 AT2G41250 AT1G53590 AT5G57900 AT4G34500 AT3G56650 AT3G18040 AT4G22010 AT1G73110 AT5G27520 AT4G31850 AT3G07700 AT4G23010 AT3G04090 AT1G70900 AT5G25460 AT4G35790 AT1G10740 AT2G16400 AT3G56260 AT1G22985 AT3G06750 AT5G66900 AT2G01650 AT5G14120 AT3G22370 AT1G69530 AT1G05790 AT2G29310 AT2G32100 AT1G30520 AT4G04620 AT5G16880 AT3G21250 AT4G30390 AT5G02490 AT5G01450 AT5G39210 AT2G06850 AT5G57655 AT3G26100 AT5G20600
