# Supplementary material for: Genome-Wide Patterns of Arabidopsis Gene Expression in Nature
Source: PLoS Genet. 2012 Apr 19;8(4):e1002662. doi: 10.1371/journal.pgen.1002662 (PMC3330097; doi:10.1371/journal.pgen.1002662)
Supplement: Table S5 — List of genes correlated to PC2veg (upper and lower 2.5 to 5% of the quantile distributions). (DOCX) [file pgen.1002662.s009.docx]

**Table S5.** List of genes correlated to PC2^veg^ (upper and lower 2.5 to 5% of the quantile distributions).

AT5G27280 AT2G30250 AT5G52810 AT4G23750 AT5G59240 AT5G11390 AT2G45500 AT5G11800 AT1G07510 AT3G17609 AT3G22600 AT2G26690 AT3G04940 ATMG01010 AT1G17170 AT1G33440 AT4G31820 AT5G26880 AT3G54840 AT1G61740 AT5G18470 AT5G23870 AT1G01470 AT2G41000 AT5G58510 AT4G32680 AT3G21610 AT2G10950 AT1G03600 AT1G17160 ATCG00130 AT4G30890 AT4G32960 AT5G24810 AT1G14130 AT1G13340 AT1G18090 AT3G55710 AT3G57090 AT3G61220 AT1G30640 AT3G09440 AT1G22630 AT2G17290 AT3G11110 AT2G36330 ATCG00520 AT2G33430 AT3G50210 AT4G16447 AT5G15360 AT1G64140 AT4G05070 AT5G45650 AT4G36760 AT1G05560 AT1G73602 AT2G30620 AT1G08940 AT2G32190 AT2G14740 AT1G15550 AT4G25850 AT2G25080 AT1G76860 AT2G32990 AT3G16560 AT5G13750 AT1G06240 AT2G20010 AT1G47340 AT1G53030 AT4G34220 AT4G16330 AT5G51550 AT5G67340 AT1G22330 AT3G03270 AT1G15180 AT5G17010 AT2G40830 AT4G21870 AT5G52780 AT3G26470 AT1G07020 AT3G14680 AT1G19050 AT2G07674 AT4G26950 AT5G52550 AT1G51160 AT2G17190 AT4G36110 AT5G04810 AT4G00560 AT5G01350 AT3G10740 AT5G55850 AT1G77030 AT2G44420 AT5G45900 AT5G62880 AT1G47870 AT2G33480 AT5G19460 AT5G44240 AT5G57020 AT2G30100 AT4G28570 AT1G21770 AT1G54710 AT5G15230 AT2G34510 AT3G11530 AT4G39770 AT4G34730 AT3G49060 AT4G15920 AT5G41070 AT1G10700 AT1G18620 AT4G22770 AT2G26110 AT3G44110 AT3G52180 AT5G17300 AT5G40760 AT5G37930 AT3G62980 AT4G10160 AT2G03050 AT2G47000 AT3G15650 AT4G37550 AT3G10210 AT3G50880 AT2G18570 AT1G18400 AT1G02410 AT4G33780 AT4G10955 AT4G39960 AT3G09270 AT4G33150 AT5G06810 AT5G09310 AT4G15480 AT4G16540 AT1G11410 AT5G43960 AT1G29670 AT3G55510 AT5G07010 AT2G42070 AT1G62030 AT4G04800 AT4G35980 AT1G07890 AT4G32150 AT5G53330 AT1G06290 AT2G25000 AT5G61410 ATCG00490 AT1G04530 AT3G19360 AT1G65660 AT3G22240 AT5G62710 AT1G01090 AT5G41560 AT1G14210 AT5G53420 AT2G16790 AT5G02560 AT5G17450 AT4G03200 AT1G73920 AT3G16030 AT3G27380 AT1G65590 AT1G08280 AT3G23690 AT1G70810 AT5G13010 AT4G22840 AT1G80130 AT5G61510 AT5G42240 AT5G07820 AT4G28180 AT1G33040 AT3G27260 AT3G55470 AT1G69040 AT5G60540 AT5G59210 AT4G38260 AT5G46760 AT1G31660 AT3G55030 AT2G37970 AT2G16380 AT2G23150 AT3G60800 AT5G43100 AT1G76160 AT1G10920 AT2G26230 AT1G17020 AT1G64142 AT1G62880 AT2G02870 AT3G54900 AT2G43130 AT4G23210 AT4G24740 AT1G31850 AT1G32750 AT2G30520 AT3G19280 AT5G20950 AT4G26520 AT5G55200 AT3G53410 AT4G23270 AT1G79630 AT3G27570 AT2G35120 AT1G34420 AT2G02990 AT4G18130 AT5G58290 AT3G06540 AT4G18430 AT5G09930 AT1G77450 AT2G47460 AT1G09310 AT1G04970 AT1G18070 AT4G14560 AT2G28950 AT2G02810 AT5G48170 AT5G24870 AT3G27000 AT4G00550 AT3G27540 AT2G40810 AT1G69160 AT2G47420 AT1G14450 AT2G36320 AT1G02270 AT3G09735 AT2G42390 AT1G70290 AT1G26300 AT2G30950 AT1G74020 AT5G48370 AT2G14720 AT3G44190 AT2G18940 AT4G10960 AT3G10320 AT1G30440 AT3G51820 AT3G55390 AT2G18560 AT1G07450 AT5G58920 AT3G07220 AT1G29390 AT4G12690 AT3G62410 AT4G38560 AT3G07610 AT2G05630 AT4G16430 AT5G56650 AT5G04020 AT5G04910 AT5G56660 AT5G41400 AT3G09320 AT3G53230 AT2G23130 AT1G66920 AT4G29040 AT5G53560 AT4G15610 AT2G21940 AT1G08050 AT5G10270 AT1G76110 AT3G47620 AT3G14720 AT2G32910 AT1G66390 AT1G50410 AT1G54130 AT4G17530 AT4G29680 AT1G09560 AT2G37110 AT1G10840 AT1G70250 AT4G37980 AT3G54500 AT1G55280 AT1G35580 AT5G60460 AT5G63990 AT1G17970 AT5G55620 AT1G11910 ATCG00140 AT3G07260 AT2G37260 AT2G26480 AT4G22740 AT4G23150 AT3G11930 AT3G23880 AT2G34850 AT1G07790 AT3G28540 AT2G16900 AT5G04590 AT2G46410 AT3G21260 AT2G21220 AT1G73990 AT5G61020 AT3G45620 AT3G61200 AT4G11800 AT4G25650 AT1G63460 AT4G37750 AT4G05160 AT3G63310 AT2G24360 AT2G03020 AT1G62180 AT1G22700 AT4G35600 AT4G14870 AT4G34131 AT5G22060 AT3G02360 AT1G05340 AT2G02390 AT3G47120 AT1G20810 AT1G20490 AT3G21400 AT3G43210 AT3G03090 AT1G68820 AT5G54070 AT1G66140 AT4G10150 AT1G18270 AT1G17490 AT2G43950 AT3G12350 AT1G61260 AT1G22360 AT4G33540 AT5G55480 AT2G05590 AT5G22220 AT3G28210 AT5G05570 AT2G01170 AT1G37130 AT1G54570 AT5G04781 AT3G53930 AT5G64300 AT3G46130 AT5G54470 AT2G17380 AT1G07630 AT1G16720 AT1G70070 AT3G23410 AT3G54920 AT4G11890 AT4G19670 AT4G33910 AT1G70230 AT4G21160 AT5G39860 AT4G26480 AT5G51640 AT2G36380 AT4G17270 AT1G59580 AT3G27240 AT1G73220 AT4G34770 AT4G03080 AT1G02220 AT5G63140 AT3G07310 AT1G17745 AT3G07190 AT4G39660 AT2G42680 AT5G18270 AT1G24280 AT1G32940 AT5G62360 AT2G31200 AT2G22470 AT1G65900 AT4G34135 AT3G06760 AT1G61250 AT4G37370 AT1G18160 AT5G26200 AT1G18870 AT1G75460 AT5G53360 AT3G10640 AT3G56460 AT3G10350 AT1G01320 AT1G66910 AT4G16500 AT1G11180 AT5G51070 AT3G17820 AT5G23240 AT1G32870 AT5G24430 AT5G52390 AT4G00500 AT2G46310 AT4G04850 AT1G28480 AT3G09010 AT3G60620 ATCG00680 AT2G32210 AT3G48690 AT4G34250 AT3G14910 AT3G02290 AT5G49570 AT2G37640 AT5G52552 AT5G20270 AT3G54680 AT4G38860 AT5G52240 AT4G25860 AT3G46290 AT1G68620 AT5G17980 AT3G06690 AT1G56220 AT2G40980 AT1G17260 AT2G32260 AT1G31280 AT5G15400 AT5G52280 AT3G50560 AT3G46790 AT5G19470 AT4G03410 AT2G26170 AT1G75090 AT4G09820 AT1G48160 AT2G27130 AT4G34050 AT5G56540 AT3G05970 AT3G57490 AT5G48400 AT4G21470 AT2G34660 AT2G21050 AT4G30830 AT1G73380 AT1G73600 AT5G58550 AT1G71980 AT4G17486 AT5G62920 AT3G17810 AT2G42270 AT2G17200 AT5G52440 AT4G12980 AT4G16760 AT5G46240 AT4G33400 AT4G39140 AT1G26920 AT5G13950 AT3G13610 AT1G75170
