# Supplementary material for: Genome-Wide Patterns of Arabidopsis Gene Expression in Nature
Source: PLoS Genet. 2012 Apr 19;8(4):e1002662. doi: 10.1371/journal.pgen.1002662 (PMC3330097; doi:10.1371/journal.pgen.1002662)
Supplement: Table S6 — Significant differences in fitness (fruit number) associated with gene functions in fluctuating temperature (PC1veg) and water availability (PC2veg) treatments (DOCX) [file pgen.1002662.s010.docx]

**Table S6.** Significant differences in fitness (fruit number) associated with gene functions in fluctuating temperature (PC1^veg^) and water availability (PC2^veg^) treatments.

| **Gene name** | **Locus** | **Mutant line** | **PC^veg^** | **Genotype^a^** | **G x E^b^** |
| --- | --- | --- | --- | --- | --- |
| *SDG37* | At2g17900 | SALK_018048C | PC1 | 0.6747 | 0.0858 |
| *CPN10* | At1g14980 | SALK_081250C | PC1 | 0.5156 | 0.6112 |
| *TCP10* | At2g31070 | SALK_137205C | PC1 | 0.7368 | 0.7040 |
| *HSP70* | At3g12580 | SALK_038113 | PC1 | 0.1295 | 0.9246 |
| *HSP83* | At5g52640 | SALK_065166 | PC1 | 0.7323 | 0.4528 |
| *ASIL1.14* | At1g54050 | SALK_072866 | PC1 | 0.0156 | 0.1490 |
| *T1P17.2* | At4g12400 | SALK_023494 | PC1 | 0.0642 | 0.2463 |
| *ARP6* | At3g33520 | SALK_037471C | PC1 | 0.4568 | 0.3258 |
| *ROP10* | At3g48040 | SALK_018747C | PC2 | 0.8883 | 0.9549 |
| *AAO3* | At2g27150 | SALK_072361C | PC2 | 0.0001^***^ | 0.0007^**^ |
| *ATTPS5* | At4g17770 | SALK_007952 | PC2 | 0.4695 | 0.3964 |
| *GASA1* | At1g75750 | SALK_001187C | PC2 | 0.5101 | 0.7903 |
| *ALDH7B4* | At1g54100 | SALK_030725 | PC2 | 0.0013^*^ | 0.0080^*^ |
| *CIPK20* | At5g45820 | SALK_003402 | PC2 | 0.6417 | 0.8415 |

Genes identified in PC1^veg^ were subjected to either normal or fluctuating temperature, and in PC2^veg^ to well- or moderately-watered conditions. ^a^p-values for effect of mutant vs. wild-type allele, ^b^p-values for interaction of genotype (wild-type vs. mutant allele) with environmental conditions. ^*^ *p* < 0.01, ^**^ *p* < 0.001, ^***^ *p* < 0.0001.
