# Supplementary material for: Genome-Wide Patterns of Arabidopsis Gene Expression in Nature
Source: PLoS Genet. 2012 Apr 19;8(4):e1002662. doi: 10.1371/journal.pgen.1002662 (PMC3330097; doi:10.1371/journal.pgen.1002662)
Supplement: Text S1 — Fitness analysis. (DOC) [file pgen.1002662.s011.doc]

**Text S1: Fitness analysis**

We grew *A. thaliana* T-DNA insertions lines for eight genes that loaded strongly in PC1^veg^ and were associated with both maximum daily field temperatures and temperature fluctuations (see Supplementary Table S6). The T-DNA SALK insertion lines were genotyped using the oligo pairs LBb1.3 and RP for the mutant allele, and RP and LB for the wild type allele as recommended by the T-DNA primer design website http://signal.salk.edu/tdnaprimers.2.html. PCR products of ~1kb were obtained using 55^o^C as annealing temperature and 35 cycles. Homozygote plants were identified and seed was bulked up for the fitness assays.

Seeds were stratified at 4^o^C for four days in the dark, and sown in pots with wet soil (Metro-Mix 360, SUNGRO Horticulture, USA). Ten days after sowing, the seedlings were transplanted to 72-plug trays in a fully randomized design, and grown for four days at constant temperature (22^o^C). On day 14 after sowing, some trays were transferred to a condition that simulated three cycles of 35 days, according to the temperature fluctuations of our field experiment. The temperatures were linearly ramped hourly from the minimum temperature to the maximum temperature and back to the minimum temperature for any given day. As a control environment, another set of plants was grown in a conventional constant temperature for *A*. *thaliana* studies (22^o^C). Plants in both conditions were grown simultaneously in two Percival Intellus growth chambers (Percival, Percival Scientific, USA), long day 16h light/8h dark, and trays were switched between chambers every 4 days to minimize chamber effects. The experiment had 15 to 22 plants per treatment, per genotype, for a total of 375 plants. We counted total numbers of fruits once the plant was dry. We used ANOVA in JMP 5.0 (SAS, USA), with the effects of genotype (wild-type vs. mutant), environment (constant vs. fluctuating temperature), genotype-by-environment interaction, and block effect terms, according to the following model [Fruit number = genotype + environment + genotype*environment + block + ε].

We also examined T-DNA insertions lines under drought conditions for six genes that were strongly loaded on PC2^veg^ in response to two treatments: moderately watered plants and severe drought. We determined that for 27.5 g of soil in every pot, 50 mL of water are equivalent to 100% water soil capacity. Thus the addition of 25 mL or 10 mL to every pot is equivalent to 50% and 20% water soil capacity (bottom water leaking was negligible). Eleven days after sowing stratified seeds and growth in well-watered conditions, we watered the moderately-watered plants to 50% water soil capacity every 5 days, and the severe drought plants to 20% field capacity every 10 days. The two treatments, including mutants and wild type, were grown in a randomized design in a walk in growth chamber. The experiment consisted of 15-20 plants per genotype, per treatment, for a total of 288 plants. We used ANOVA with two factors, water availability, and genotype (wild type and mutant) and the interaction, plus the block effect to analyze the data similar to the model above.

Of the nine genes with strong loadings on PC1^veg^, there were no significant effects of genotype (p < 0.13), significant effects due to environment (p < 0.019), no significant interaction of these two factors (p < 0.58), and a significant block effect (p < 0.016)[see Supplementary Table S6]. This suggests that fluctuating temperature by itself does not decrease fitness in the selected mutants, but possibly interacts with a more complex combination of environmental factors.

We also tested a set of mutant lines corresponding to six genes with strong loading in PC2^veg^ that was subjected to reduced water availability (from 50% soil water capacity every five days to 20% soil water capacity every ten days). In the ANOVA we found significant effects from all factors, including genotype (p < 0.0004), environment (p < 0.0001), and the interaction term (p < 0.010), and no significant block effect (p < 0.11). The genes that appear to contribute most to the interaction effect are *AAO3* (p < 0.0007) and *ALDH7B4* (p < 0.0080)[see Table Supplementary Table S6 and Supplemental Figure 4].
